# Natural and synthetic 2-oxoglutarate derivatives are substrates for oncogenic variants of human isocitrate dehydrogenase 1 and 2

**DOI:** 10.1016/j.jbc.2023.102873

**Published:** 2023-01-05

**Authors:** Xiao Liu, Raphael Reinbold, Shuang Liu, Ryan A. Herold, Patrick Rabe, Stéphanie Duclos, Rahul B. Yadav, Martine I. Abboud, Sandrine Thieffine, Fraser A. Armstrong, Lennart Brewitz, Christopher J. Schofield

**Affiliations:** 1 Chemistry Research Laboratory, Department of Chemistry and the Ineos Oxford Institute for Antimicrobial Research, University of Oxford, Oxford, United Kingdom; 2 Inorganic Chemistry Laboratory, Department of Chemistry, University of Oxford, Oxford, United Kingdom; 3 Evotec (UK) Ltd, Abingdon, United Kingdom; 4 Evotec (US) Inc., Princeton, NJ, USA

**Keywords:** isocitrate dehydrogenase, IDH, cancer metabolism-related IDH mutations, 2-oxoacids, α-ketoacids, 2-oxoglutarate, α-ketoglutarate, 2OG, (*R*)-2-hydroxyglutarate, 2HG, alternative substrates, epigenetics, acute myeloid leukemia, 2OG, 2-oxoglutarate, 2HG, D-2-hydroxyglutarate, AML, acute myeloid leukemia, AspH, aspartate/asparagine-β-hydroxylase, CPMG, Carr–Purcell–Meiboom–Gill, FIH, factor inhibiting hypoxia inducible factor-α, FNR, ferredoxin NADP+-reductase, IDH, isocitrate dehydrogenase, ITO, indium tin oxide, PGE, pyrolytic graphite edge, NOG, *N*-oxalylglycine, TCA, tricarboxylic acid

## Abstract

Variants of isocitrate dehydrogenase (IDH) 1 and 2 (IDH1/2) alter metabolism in cancer cells by catalyzing the NADPH-dependent reduction of 2-oxoglutarate (2OG) to (*2R*)-hydroxyglutarate. However, it is unclear how derivatives of 2OG can affect cancer cell metabolism. Here, we used synthetic C3- and C4-alkylated 2OG derivatives to investigate the substrate selectivities of the most common cancer-associated IDH1 variant (R132H IDH1), of two cancer-associated IDH2 variants (R172K IDH2, R140Q IDH2), and of WT IDH1/2. Absorbance-based, NMR, and electrochemical assays were employed to monitor WT IDH1/2 and IDH1/2 variant-catalyzed 2OG derivative turnover in the presence and absence of 2OG. Our results reveal that 2OG derivatives can serve as substrates of the investigated IDH1/2 variants, but not of WT IDH1/2, and have the potential to act as 2OG-competitive inhibitors. Kinetic parameters reveal that some 2OG derivatives, including the natural product 3-methyl-2OG, are equally or even more efficient IDH1/2 variant substrates than 2OG. Furthermore, NMR and mass spectrometry studies confirmed IDH1/2 variant-catalyzed production of alcohols in the cases of the 3-methyl–, 3-butyl–, and 3-benzyl–substituted 2OG derivatives; a crystal structure of 3-butyl-2OG with an IDH1 variant (R132C/S280F IDH1) reveals active site binding. The combined results highlight the potential for (i) IDH1/2 variant-catalyzed reduction of 2-oxoacids other than 2OG in cells, (ii) modulation of IDH1/2 variant activity by 2-oxoacid natural products, including some present in common foods, (iii) inhibition of IDH1/2 variants *via* active site binding rather than the established allosteric mode of inhibition, and (iv) possible use of IDH1/2 variants as biocatalysts.

Isocitrate dehydrogenases (IDHs) catalyze the NADP^+^- or NAD^+^-dependent oxidative decarboxylation of D-isocitrate to 2-oxoglutarate (2OG, α-ketoglutarate) and CO_2_, and NADPH or NADH (Fig. 1*A*) (1). In humans, NAD^+^-dependent IDH3 is part of the tricarboxylic acid (TCA) cycle, whereas NADP^+^-dependent dimeric IDH1 and 2 (IDH1/2) catalyze the same reaction, but apparently independent of the TCA cycle. The most common mutations in metabolic enzymes in cancer are somatic mutations in the *IDH1/2* genes which are found in >80% of low-grade glioma and ∼20 to ∼30% of acute myeloid leukemia (AML) patients (2, 3, 4). These active site variants still catalyze the conversion of isocitrate to 2OG, albeit at a reduced rate, but have a substantially enhanced ability to reduce 2OG to the oncometabolite (*R*)-2-hydroxyglutarate (2HG), a reaction only catalyzed very poorly by WT IDH1/2 (Fig. 1*B*) (3, 5, 6). The gain of function activity of the IDH1/2 variants results in substantially elevated intracellular 2HG levels (and increased NADP^+^ levels), which are proposed to promote tumorigenesis (in combination with mutation to oncogenes others than those for IDH1/2) (3, 4, 5, 6, 7).

**Figure 1:**
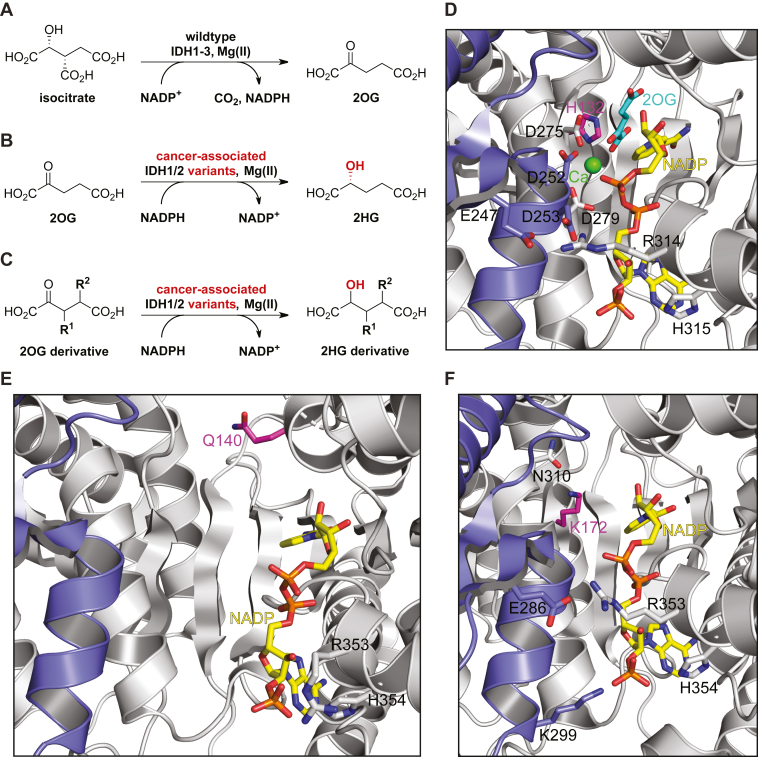
**Isocitrate dehydrogenase catalysis.***A*, WT IDH1-3 catalyze the Mg(II)-dependent conversion of isocitrate to 2OG and CO_2_ coupled with conversion of the cosubstrate NADP^+^ (or NAD^+^) reduction to NADPH (or NADH); (*B*) cancer-associated IDH variants, *e.g.* R132H IDH1, R132C IDH1, R172K IDH2, and R140Q IDH2, catalyze the stereospecific reduction of 2OG to (*R*)-2-hydroxyglutarate (2HG) coupled with conversion of NADPH to NADP^+^. Note that WT IDH1/2 do not efficiently catalyze the reduction of 2OG to 2HG (3, 5); (*C*) potential reaction of cancer-associated IDH1/2 variants with 2OG derivatives; (*D*) view of the R132H IDH1 active site in a closed conformation in complex with Ca(II) (*green*), 2OG (*teal*), and NADP^+^ (*yellow*), the R132H variation is in *magenta*; two R132H IDH1 molecules (*gray* and *lavender*) interact with each other and form the catalytically active dimer (PDB ID: 3INM (54)); (*E*) view of the R140Q IDH2 active site in complex with NADP^+^ (*yellow*) in an open conformation; the R140Q variation is in *magenta*; two R140Q IDH2 molecules (*gray* and *lavender*) interact with each other and form the catalytically active dimer (PDB ID: 5SVO (75)); (*F*) view of the R172K IDH2 active site in complex with NADP^+^ (*yellow*) in a closed conformation; the R172K variation, which corresponds to R132 in IDH1, is in *magenta*; two R172K IDH2 molecules (*gray* and *lavender*) interact with each other and form the catalytically active dimer (PDB ID: 5SVN (75)). 2OG, 2-oxoglutarate; IDH, isocitrate dehydrogenase.

Clinically useful IDH1/2 variant inhibitors have been developed which decrease 2HG levels and which appear efficacious for (at least) AML treatment (*e.g.* ivosidenib (8), enasidenib (9)). Interestingly, all the potent IDH1/2 variant inhibitors active *in vivo* bind allosterically at the IDH dimer interface; their selectivity for IDH variant inhibition arises (at least in part) due to disruption of Mg(II) binding which has a larger effect on binding of 2OG than the isocitrate:Mg(II) complex WT substrate (10). Resistance to the allosterically binding IDH variant inhibitors has emerged *inter alia via* mutation(s) at the dimer interface, potentially compromising the use of these inhibitors for clinical applications (11, 12). Thus, there is interest in developing new types of IDH variant inhibitors, including those binding at the active site (Fig. 1*C*).

2OG is of fundamental importance in metabolism, playing key roles in the TCA cycle, amino acid/protein biosynthesis, and multiple other processes (13, 14, 15). 2OG and Fe(II)-dependent oxygenases couple substrate oxidation with 2OG decarboxylation to give succinate and CO_2_; they have important functions, for example in hypoxia signaling, DNA/RNA damage repair, extracellular matrix biosynthesis, and lipid metabolism (16). Elevated levels of 2HG in IDH1/2 variant–bearing cells are proposed to help promote tumorigenesis by inhibiting 2OG oxygenases, including ten-eleven-translocation methylcytosine oxygenases, which catalyze methylcytosine oxidations that can result in demethylation, and the Jumonji-C domain *N*-methyl lysine demethylases (JmjC KDMs), which catalyze the demethylation of *N*^ε^-mono–, di-, and tri-methylated lysine residues or *N*-methylated arginine residues in histones (3, 4, 5). Studies with isolated recombinant human 2OG oxygenases have revealed that 2HG inhibits by binding to the active site in a 2OG-competitive manner (3, 4, 5, 17). Similarly, elevated succinate and fumarate levels associated with mutations to the genes encoding for succinate dehydrogenase and fumarate hydratase are proposed to alter biological processes *via* inhibiting 2OG oxygenases and potentially other enzymes (18, 19). Recent work has highlighted the potential of 2-oxoacids other than 2OG, including naturally occurring compounds, to act as both as inhibitors and substrates of human 2OG oxygenases (20, 21, 22, 23, 24).

Considering the widespread abundance of 2-oxoacid and alcohol couples in cells, it seems likely that cancer-associated IDH1/2 variants affect metabolism in ways other than catalyzing the reduction of 2OG to 2HG. For example, recent evidence suggests that extracellular 2HG is taken up by CD8-positive T cells in which it inhibits lactate dehydrogenase, thus altering glucose metabolism resulting in decreased proliferation, decreased cytokine production, and a decreased ability to kill target cells (25). Some 2-oxoacid and 2OG derivatives have been shown to be poor substrates of pig and rat WT IDH (obtained from homogenized pig heart and rat liver, respectively) (13, 26, 27, 28, 29, 30, 31, 32, 33, 34, 35); however, the substrate selectivity of human oncogenic IDH1/2 variants with 2-oxoacids other than 2OG has not yet been investigated. Such studies are of interest because some 2OG derivatives are present in biology, including, for example, C3- and C4-alkylated 2OG derivatives in human food (Fig. 1*C*) (36, 37, 38, 39). Here, we describe studies on the selectivity of cancer-associated IDH1/2 variants with respect to 2OG derivatives, the results of which support the possibility of active site binding–mediated inhibition of IDH1/2 variants, highlight the possibility of non-2OG substrates/inhibitors of IDH1/2 variants, and suggest IDH1/2 variants have potential as biocatalysts.

## Results

### 2-Oxoacid natural products other than 2OG are substrates of cancer-associated IDH1/2 variants

Naturally-occurring 2-oxoacids and structurally related small molecules, including TCA cycle intermediates, were initially investigated as potential inhibitors of recombinant human R132H IDH1, R172K IDH2, and R140Q IDH2 with the aim of exploring potential active site–binding inhibitors (Fig. 1). Reported absorbance-based assays, which monitor conversion of NADPH to NADP^+^ and which were performed in the presence of 2OG as a substrate (1, 10), were employed to investigate 43 small molecules for IDH1/2 variant inhibition. The results reveal that of all the 43 small molecules investigated for inhibition, only *N*-oxalylglycine (NOG) efficiently inhibited all three tested cancer-associated IDH1/2 variants in the presence of equimolar amounts of 2OG (Table S1). NOG is a natural product closely related to 2OG (the 2OG methylene unit adjacent to the ketone is substituted for an NH group in NOG) (40); it is a reported 2OG-competitive inhibitor of R132H IDH1 (41) and of multiple 2OG oxygenases (42).

The 43 molecules were investigated as potential substrates for the IDH1/2 variants using absorbance assays in the absence of 2OG. None of the tested molecules were, at least, efficient substrates for R132H IDH1 or R172K IDH2 (Table S2), but the human metabolite DL-4-hydroxy-2OG (43, 44) and the plant and bacterial metabolite DL-4-hydroxy-4-methyl-2OG (parapyruvate) (45, 46) were substrates for R140Q IDH2, showing ∼20% substrate activity compared to 2OG after incubation for 20 min (Table S2). These observations are of interest because they raise the possibility that human 2-oxoacid metabolites other than 2OG may be substrates of R140Q IDH2 and potentially other IDH1/2 variants in a cellular context and that active site–binding 2OG derivatives/mimics might be developed that are selective inhibitors of cancer-associated IDH1/2 variants.

The potential of the compounds as inhibitors or substrates of isolated recombinant human WT IDH1/2 was investigated in the presence or absence of DL-isocitrate. No evidence for efficient WT IDH1/2 inhibition (>30%) was accrued in the presence of equimolar amounts of DL-isocitrate and the potential small-molecule inhibitors (Table S3). In the absence of DL-isocitrate, only the DL-isocitric acid-derived lactone showed evidence for turnover (∼10% compared to DL-isocitrate), likely due to *in situ* hydrolysis (Table S4).

### IDH1/2 variants accept synthetic 2OG derivatives as substrates

The observation that DL-4-hydroxy-2OG and DL-4-hydroxy-4-methyl-2OG are R140Q IDH2 substrates raised the possibility that 2OG derivatives might selectively bind at the active site and act as substrates or inhibitors of IDH1/2 variants. Thus, we investigated the potential activities of 32 synthetic 2OG derivatives (22) as substrates for R132H IDH1, R172K IDH2, and R140Q IDH2, using absorbance assays (Table 1). Note that the chiral 2OG derivatives were racemates. We have previously shown that some of these 2OG derivatives are substrates and/or inhibitors of the human 2OG oxygenases aspartate/asparagine-β-hydroxylase (AspH) (47, 48, 49) and factor-inhibiting hypoxia inducible factor-α (FIH) (50, 51, 52), which catalyze the oxidations of specific residues in epidermal growth factor–like domains and ankyrin repeat domains/other proteins, respectively (22, 23). Crystal structures of some of the 2OG derivatives, which were alternative substrates and/or inhibitors, in complex with AspH and FIH implies stereoselectivity in their binding modes (22, 23).

**Table 1:** Synthetic C3-/C4-substituted 2OG derivatives are substrates of cancer-associated IDH1/2 variants

Entry	^a^2OG derivative	IDH1/2 variant	^b^Activity [%]	Entry	^a^2OG derivative	IDH1/2 variant	^b^Activity [%]	Entry	^a^2OG derivative	IDH1/2 variant	^b^Activity [%]
A		^c^R132H	100	^f^L		^c^R132H	<10	^h^W		^c^R132H	20 ± 2
		^d^R172K	100			^d^R172K	<10			^d^R172K	<10
		^e^R140Q	100			^e^R140Q	<10			^e^R140Q	24 ± 6
B		^c^R132H	275 ± 15	M		^c^R132H	52 ± 5	X		^c^R132H	79 ± 10
		^d^R172K	119 ± 7			^d^R172K	14 ± 3			^d^R172K	14 ± 8
		^e^R140Q	971 ± 28			^e^R140Q	526 ± 23			^e^R140Q	394 ± 13
C		^c^R132H	157 ± 12	N		^c^R132H	<10	^i^Y		^c^R132H	<10
		^d^R172K	32 ± 3			^d^R172K	<10			^d^R172K	<10
		^e^R140Q	731 ± 26			^e^R140Q	15 ± 1			^e^R140Q	59 ± 6
D		^c^R132H	310 ± 49	O		^c^R132H	<10	^j^Z		^c^R132H	<10
		^d^R172K	37 ± 10			^d^R172K	<10			^d^R172K	<10
		^e^R140Q	857 ± 58			^e^R140Q	25 ± 5			^e^R140Q	<10
E		^c^R132H	410 ± 22	P		^c^R132H	<10	AA		^c^R132H	<10
		^d^R172K	32 ± 3			^d^R172K	<10			^d^R172K	<10
		^e^R140Q	987 ± 72			^e^R140Q	14 ± 3			^e^R140Q	<10
F		^c^R132H	75 ± 3	Q		^c^R132H	<10	^k^AB		^c^R132H	<10
		^d^R172K	<10			^d^R172K	<10			^d^R172K	<10
		^e^R140Q	288 ± 19			^e^R140Q	35 ± 3			^e^R140Q	<10
G		^c^R132H	124 ± 3	R		^c^R132H	<10	^l^AC		^c^R132H	11 ± 3
		^d^R172K	<10			^d^R172K	<10			^d^R172K	<10
		^e^R140Q	233 ± 45			^e^R140Q	15 ± 5			^e^R140Q	14 ± 5
H		^c^R132H	141 ± 7	S		^c^R132H	<10	AD		^c^R132H	<10
		^d^R172K	13 ± 5			^d^R172K	<10			^d^R172K	<10
		^e^R140Q	824 ± 57			^e^R140Q	<10			^e^R140Q	<10
I		^c^R132H	106 ± 7	T		^c^R132H	<10	AE		^c^R132H	<10
		^d^R172K	<10			^d^R172K	<10			^d^R172K	<10
		^e^R140Q	731 ± 42			^e^R140Q	12 ± 2			^e^R140Q	<10
J		^c^R132H	112 ± 7	U		^c^R132H	<10	AF		^c^R132H	<10
		^d^R172K	11 ± 3			^d^R172K	<10			^d^R172K	<10
		^e^R140Q	140 ± 14			^e^R140Q	<10			^e^R140Q	<10
K		^c^R132H	19 ± 3	^g^V		^c^R132H	43 ± 7	AG		^c^R132H	<10
		^d^R172K	<10			^d^R172K	25 ± 2			^d^R172K	<10
		^e^R140Q	179 ± 14			^e^R140Q	623 ± 25			^e^R140Q	<10

^a^ 2OG derivatives were prepared from cyanosulfur ylides as racemic mixtures as reported (22).

^b^ %-substrate conversion after 20 min incubation; absorbance assays were performed as described in the Experimental procedures section.

^c-e^ using 50 μM NADPH and (c) 0.03 μM R132H IDH1 and 1.5 mM 2OG derivative, (d) 0.03 μM R172K IDH2 and 1.0 mM 2OG derivative, or (e) 0.03 μM R140Q IDH2 and 6.0 mM 2OG derivative in reaction buffer (100 mM Tris, pH 8.0, 0.005%_v/v_ tween-20, 0.1 mg/mL BSA, 0.2 mM DTT, 10 mM MgCl_2_, 150 mM NaCl).

^f^ note, **11** is an inhibitor (see main text).

^g^ mixture of racemic diastereomers, dr (*cis*:*trans*) = 2.5:1.

^h^ mixture of racemic diastereomers, dr (*cis*:*trans*) = 1:1.

^i^ mixture of racemic diastereomers, dr (*cis*:*trans*) = 2.5:1.

^j^ mixture of diastereomers, dr (*trans*:*cis*) = 5:1.

^k^ mixture of diastereomers, dr (*cis*:*trans*) = 10:1.

^l^ (±)-(*2-exo,3-endo*)-diastereomer.

Results are a mean of four technical replicates (n = 4; mean ± SD).

**Figure 2:**
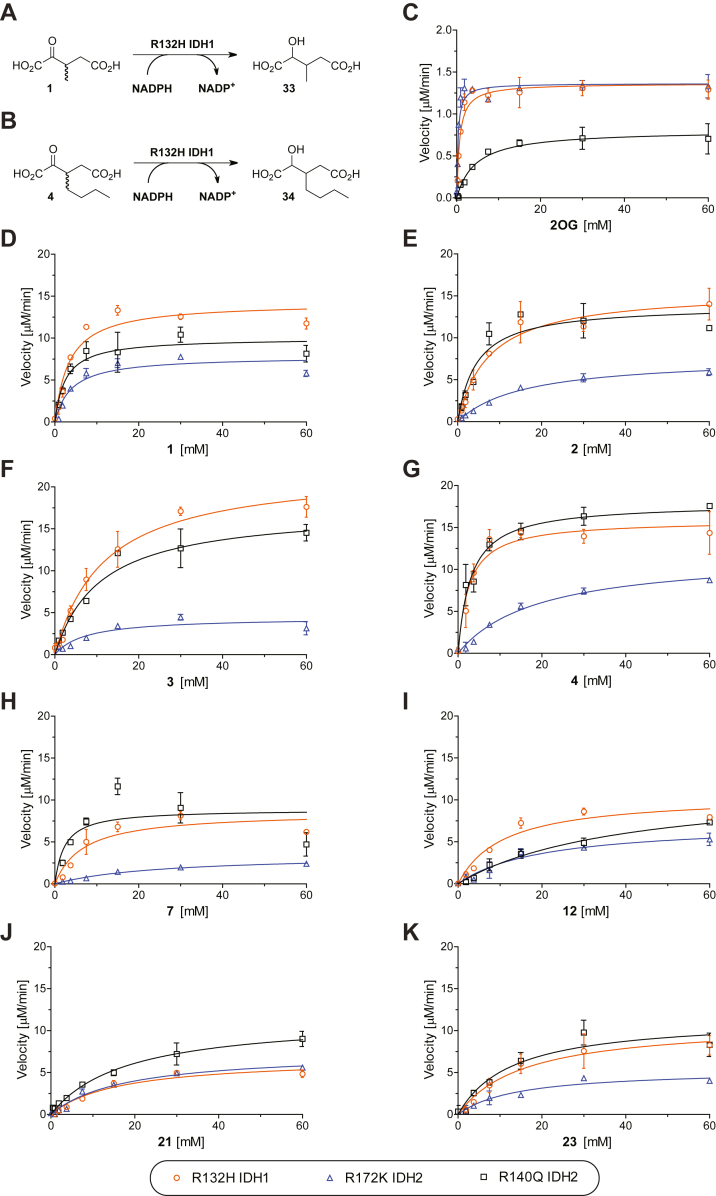
**Kinetic parameters for the IDH1/2 variant-catalyzed reduction of 2OG derivatives.***A* and *B*, the R132H IDH1 variant catalyzes the reduction of (*A*) 3-methyl-2OG (**1**) giving 2-hydroxy-3-methylglutarate **(33**) and (b) 3-butyl-2OG (**4**) giving 3-butyl-2-hydroxyglutarate (**34**) as confirmed by NMR and/or MS analyses (Fig. S1); *C*–*K*, $K_{m}^{app}$- values of R132H IDH1 (*orange circles*), R172K IDH2 (*blue triangles*), and R140Q IDH2 (*black boxes*) for (*C*) 2OG, (*D*) **1**, (*E*) **2**, (*F*) **3**, (*G*) **4**, (*H*) **7**, (*I*) **12**, (*J*) **21**, and (*K*) **23**. IDH1/2 variant assays were performed as described in the Experimental procedures section, results are a mean of two independent runs, each composed of technical duplicates (n = 2; mean ± SD). 2OG, 2-oxoglutarate.

The results reveal that 2OG derivatives **1**-**9** and **12** are efficient substrates (>50% conversion compared with 2OG control incubations) of R132H IDH1 and that **1**-**10**, **12**, **21**, **23**, and **24** are substrates for R140Q IDH2 (Table 1). Only 3-methyl-2OG (**1**) was an efficient substrate for R172K IDH2 (Table 1, entry B). Interestingly, 3-methyl-2OG (**1**) and 4-methyl-2OG (**12**) are natural products reported to be present in human food (36, 37, 38, 39). To validate the assumption that NADPH consumption reflects 2OG derivative reduction, we carried out NMR and MS studies in the case of R132H IDH1 and **1** (Fig. S1). The results showed that R132H IDH1 (and by analogy R172K IDH2 and R140Q IDH2) catalyzes reduction of **1** to 2-hydroxy-3-methylglutarate (Figs. 2 and S1). Analysis of the ^1^H NMR spectrum of the reaction mixture of **1** incubated with R132H IDH1 implies that 2-hydroxy-3-methylglutarate is produced as a single diastereomer (Fig. S1). Although the NMR studies do not inform on whether this is a single enantiomer, the stereoselective reduction of 2OG to give exclusively *2R*-2HG implies that this is likely the case, as supported by evidence for, at least, preferred reaction of a single enantiomer of **1** with R132H IDH1 (see below).

By contrast with the substrate analog turnover activity observed with the IDH1/2 variants, the absorbance assay results imply that WT IDH1/2 do not catalyze the reduction of the tested 2OG derivatives, in accord with the observation that 2OG is not an efficient substrate of WT IDH1/2 (Table S5).

Some of the tested 2OG derivatives displayed equal or better turnover with the IDH1/2 variants compared to 2OG, *e.g.* the conversion of the R132H IDH1-catalyzed reduction of **4** was approximately fourfold higher than that of 2OG (Table 1, entries A and E). In general, the 2OG derivatives bearing a substituent at the C3 position appear to be more efficient substrates than the C4-substituted regioisomers, *e.g.* **1** is a more efficient substrate than **12** (Table 1, entries B and M). In accord with this observation, 2OG derivatives in which C3 and C4 are part of a carbocycle are less efficient substrates of R140Q IDH2 and R132H IDH1 than those with a corresponding aliphatic C3 substituent but are more efficient substrates than those with a corresponding aliphatic C4 substituent, *e.g.* the cyclobutane derivative **23** is less efficient than C3 ethyl-substituted **2** but is more efficient than C4 ethyl-substituted **14** (Table 1, entries C, O, and X).

Although a variety of substituents at the 2OG C3 position are tolerated as substrates by R140Q IDH2 and R132H IDH1 (*e.g.* methyl, ethyl, propyl, butyl; Table 1, entries B-E), it appears that, in general, conformationally flexible aliphatic C3 substituents are more efficiently turned over than rigid and/or bulky C3 substituents, *e.g.* C3 butyl-substituted **4** is ∼3- to ∼4-fold more efficient substrate for R140Q IDH2 and R132H IDH1 than *tert*-butyl group bearing **5** or phenyl group bearing **6** (Table 1, entries E-G). A notable exception is the apparent relatively efficient reduction of 2OG derivatives **21** and **23** (and to a lesser extent **24**) as catalyzed by R140Q IDH2 (Table 1, entries V, X, and Y). This observation shows that, at least for R140Q IDH2, the active site can accommodate conformationally constrained 2OG derivatives. Further, R140Q IDH2, and in some cases R132H IDH1, can apparently accommodate 2OG derivatives with relatively sterically bulky substituents at C3 and C4 (*e.g.* **3**–**9**; Table 1). Future detailed structure activity relationship studies following up on these observations are of interest with respect to scoping the substrate and inhibitor selectivity of IDH variants.

In general, R140Q IDH2 appears to react more efficiently with 2OG and the tested 2OG derivatives than R132H IDH1, which both react much more efficiently than R172K IDH2. This trend may reflect the different concentrations of 2OG derivatives employed in the different assays, *i.e.*, 1.5 mM for R132H IDH1, 1.0 mM for R172K IDH2, and 6.0 mM for R140Q IDH2; note that different 2OG concentrations were employed to account for the observed differences in the $K_{m}^{app}$-values (see below). More detailed kinetic studies were thus performed with some of the more efficient substrates.

### Kinetic analyses

Kinetic analyses of R132H IDH1, R172K IDH2, and R140Q IDH2 were performed with selected 2OG derivatives, *i.e.* **1**-**4**, **7**, **12**, **21**, and **23**. Michaelis constants ($K_{m}^{app}$) and maximum velocities ($v_{\max}^{app}$) were determined using absorbance assays under the same conditions, *i.e.* 0.03 μM IDH1/2 variant and 50 μM NADPH in reaction buffer (100 mM Tris, pH 8.0, 0.005%_v/v_ tween-20, 0.1 mg/mL bovine serum albumin (BSA), 0.2 mM DTT, 20 mM MgCl_2_, 150 mM NaCl) (Fig. 2); $k_{cat}^{app}$-values (turnover numbers) were calculated from $v_{\max}^{app}$-values assuming all the enzymes in the assays were active. Note also that, since the synthetic substrates were tested as racemic mixtures, there is potential for stereoselectivity in the reactions, with respect to both reduction and inhibition, including that one enantiomer may be a substrate and the other an inhibitor. Thus, care should be taken in interpreting the values of the kinetic parameters. Nonetheless, the results imply clear differences in the selectivities of the IDH variants; further, only 2OG derivative **11** was found to inhibit IDH variants under the tested conditions (as described below).

The $k_{cat}^{app}$-values of R132H IDH1, R172K IDH2, and R140Q IDH2 for 2OG are in the same range ($k_{cat}^{app}$ ∼ 0.5–0.7 s^-1^; Table 2, entry A). By contrast, the R140Q IDH2 $K_{m}^{app}$-value for 2OG is ∼6- and ∼14-fold higher than that for R132H IDH1 and R172K IDH2, respectively, indicating a lower affinity of the R140Q IDH2 variant for 2OG. The R132H IDH1 $K_{m}^{app}$-value for 2OG is in the range of values previously reported using absorbance assays (10, 53, 54). Comparison of $k_{cat}$/ $K_{m}$ values implies 2OG is ∼21-fold and ∼9-fold less efficient substrate for R140Q IDH2 than R172K IDH2 and R132H IDH1, respectively (Table 2, entry A). To validate our IDH1/2 variant absorbance results, we determined the R132H IDH1 variant $K_{m}^{app}$-value for 2OG using an orthogonal electrochemical assay (55), which employs similar reaction conditions, however, in a nanoconfined environment. The electrochemical assays results gave a similar R132H IDH1 $K_{m}^{app}$ for 2OG compared to the absorbance assay ($K_{m}^{app}$ ∼ 0.6 mM; Fig. S2).

**Table 2:** Steady-state kinetic parameters of cancer-associated IDH1/2 variants for 2OG and selected 2OG derivatives

Entry	2OG derivative	^a,b^Parameter	R132H IDH1	R172K IDH2	R140Q IDH2
A		$k_{cat}^{app}$ [s^-1^]	0.72 ± 0.04	0.72 ± 0.04	0.49 ± 0.04
		$K_{m}^{app}$ [mM]	0.85 ± 0.19	0.35 ± 0.04	5.1 ± 1.2
		$k_{cat}$**/**$K_{m}$ [mM^-1^·s^-1^]	0.85 ± 0.22	2.1 ± 0.3	0.10 ± 0.03
B		$k_{cat}^{app}$ [s^-1^]	6.9 ± 0.4	4.8 ± 0.2	5.4 ± 0.3
		$K_{m}^{app}$ [mM]	3.7 ± 0.1	4.2 ± 0.4	2.0 ± 0.8
		$k_{cat}$**/**$K_{m}$ [mM^-1^·s^-1^]	1.9 ± 0.1	1.2 ± 0.1	2.8 ± 1.1
C		$k_{cat}^{app}$ [s^-1^]	8.3 ± 0.6	3.9 ± 0.2	8.6 ± 0.4
		$K_{m}^{app}$ [mM]	8.5 ± 1.2	15 ± 1	5.0 ± 0.5
		$k_{cat}$**/**$K_{m}$ [mM^-1^·s^-1^]	0.99 ± 0.16	0.26 ± 0.03	1.7 ± 0.2
D		$k_{cat}^{app}$ [s^-1^]	12 ± 1	2.7 ± 0.2	8.9 ± 0.8
		$K_{m}^{app}$ [mM]	13 ± 1	11 ± 5	9.4 ± 2.3
		$k_{cat}$**/**$K_{m}$ [mM^-1^·s^-1^]	0.98 ± 0.06	0.24 ± 0.11	0.95 ± 0.25
E		$k_{cat}^{app}$ [s^-1^]	9.2 ± 0.4	7.8 ± 1.8	9.7 ± 0.4
		$K_{m}^{app}$ [mM]	5.7 ± 3.3	16 ± 5	3.8 ± 0.8
		$k_{cat}$**/**$K_{m}$ [mM^-1^·s^-1^]	1.6 ± 1.0	0.50 ± 0.19	2.6 ± 0.6
F		$k_{cat}^{app}$ [s^-1^]	5.7 ± 0.6	1.9 ± 0.2	7.2 ± 0.6
		$K_{m}^{app}$ [mM]	7.2 ± 0.5	25 ± 1	7.5 ± 3.6
		$k_{cat}$**/**$K_{m}$ [mM^-1^·s^-1^]	0.80 ± 0.10	0.079 ± 0.007	0.97 ± 0.48
G		$k_{cat}^{app}$ [s^-1^]	5.3 ± 0.4	3.9 ± 0.3	6.0 ± 0.5
		$K_{m}^{app}$ [mM]	12 ± 1	25 ± 5	38 ± 3
		$k_{cat}$**/**$K_{m}$ [mM^-1^·s^-1^]	0.44 ± 0.06	0.16 ± 0.04	0.16 ± 0.02
^c^H		$k_{cat}^{app}$ [s^-1^]	3.4 ± 0.3	3.7 ± 0.2	8.0 ± 0.6
		$K_{m}^{app}$ [mM]	15 ± 2	16 ± 1	23 ± 7
		$k_{cat}$**/**$K_{m}$ [mM^-1^·s^-1^]	0.23 ± 0.04	0.23 ± 0.02	0.35 ± 0.11
I		$k_{cat}^{app}$ [s^-1^]	5.7 ± 0.4	3.0 ± 0.2	5.8 ± 0.6
		$K_{m}^{app}$ [mM]	12 ± 5	16 ± 2	14 ± 2
		$k_{cat}$**/**$K_{m}$ [mM^-1^·s^-1^]	0.47 ± 0.21	0.19 ± 0.03	0.43 ± 0.08

^a^ Mean of two independent runs, each composed of technical duplicates (n = 2; mean ± SD).

^b^ IDH1/2 variant assays monitoring NADPH turnover were performed as described in the Experimental procedures section using 0.03 μM IDH1/2 variant and 50 μM NADPH in reaction buffer (100 mM Tris, pH 8.0, 0.005%_v/v_ tween-20, 0.1 mg/mL BSA, 0.2 mM DTT, 20 mM MgCl_2_, 150 mM NaCl).

^c^ mixture of racemic diastereomers, dr (*cis*:*trans*) = 2.5:1.

Approximately, a 5 to 20-fold increase in the IDH1/2 variant $k_{cat}^{app}$-values was observed for the most efficient synthetic 2OG derivatives studied, *i.e.* **1**-**4**, **7**, **12**, **21**, and **23**, compared to 2OG itself (Table 2). While all the $k_{cat}^{app}$-values were substantially higher than for 2OG, their relative order varied with the IDH1/2 variant. For example, the $k_{cat}^{app}$-value of R172K IDH2 for C3 butyl-substituted **4** was substantially greater than that for C3 propyl-substituted **3** ($k_{cat}^{app}$ ∼ 7.8 s^−1^ and ∼ 2.7 s^−1^ for **4** and **3**, respectively; Table 2, entries D and E), whereas the R140Q IDH2 and R132H IDH1 $k_{cat}^{app}$-values for **3** and **4** were similar. These observations highlight the potential of IDH variants for use in biocatalysis.

In general, the variations in the $K_{m}^{app}$-values of the three cancer-associated IDH1/2 variants for the investigated 2OG derivatives are larger than the variations in the $k_{cat}^{app}$-values. The $K_{m}^{app}$-values for R132H IDH1 and R172K IDH2 with **1**, **3**, **21**, and **23** are in the same range, whereas those for R132H IDH1 with **2**, **4**, **7**, and **12** are about twofold lower than those for R172K IDH2, suggesting that R132H IDH1 may have a higher affinity for at least some 2OG derivatives than R172K IDH2 (Table 2). Interestingly, this observation contrasts with the observation that the R172K IDH2 $K_{m}^{app}$ for 2OG is about twofold lower than that for R132H IDH1 (Table 2, entry A). Considering that the R132H IDH1 and R172K IDH2 variations occur at the analogous active site residue (Fig. 1), these results imply other residues are important in binding of 2OG and its derivatives; conformational changes are likely important in IDH catalysis (1, 10). Note that the R132H IDH1 and R172K IDH2 $K_{m}^{app}$-values for the 2OG derivatives are all at least 4-fold greater than for 2OG and can be up to 70-fold greater, as observed with **7** and **12** for R172K IDH2 (Table 2, entries F and G). These results suggest that there is scope for improvements in substrate binding with respect to biocatalytic applications of IDH variants.

Carr–Purcell–Meiboom–Gill (CPMG) NMR spectroscopy (56) was used to investigate whether selected 2OG derivatives (*i.e.* **1**, **4**, **7**, **12**) bind to R132H IDH1 in a similar manner as 2OG; R132H IDH1 was chosen for CPMG studies due to its high yielding production, affording sufficient protein for NMR. Titrating recombinant R132H IDH1 into a 2OG/2OG derivative solution resulted in preferential depletion of the 2OG signals (∼80% signal depletion), indicating 2OG binds more efficiently than the 2OG derivatives at equimolar concentrations (Fig. S3). Signal depletion was also observed for the R132H IDH1 substrates **1**, **4**, and **7**, and less efficiently for **12** (Fig. S3), consistent with the ∼14-fold higher $K_{m}^{app}$ than 2OG (Table 2, entry G); note, binding strength/$K_{m}^{app}$-values do not necessarily correlate with catalytic efficiency. Importantly, the NMR studies reveal ∼50% signal depletion, within experimental error, for the tested C3 alkyl-substituted 2OG derivatives, whereas ∼80% signal depletion was observed for 2OG (Fig. S3). This observation is consistent with the proposal that only one enantiomer of C3 alkyl-substituted 2OG derivatives binds to the IDH variant active site.

While the R132H IDH1 and R172K IDH2 $K_{m}^{app}$-values for 2OG derivatives are higher than that obtained for 2OG, some of the R140Q IDH2 $K_{m}^{app}$-values for 2OG derivatives are more similar to that of 2OG (Table 2). For example, while the R140Q IDH2 $K_{m}^{app}$-value for 4-methyl-2OG (**12**) is ∼8-fold greater than for 2OG, the $K_{m}^{app}$-values for the racemic C3-substituted 2OG derivatives **2**, **4**, and **7** are in the same range as for 2OG (3.8–7.5 mM, compared to ∼5.1 mM for 2OG; Table 2). Remarkably, the R140Q IDH2 $K_{m}^{app}$-value for 3-methyl-2OG (**1**), a natural product present in honey (36), is more than two-fold lower than the value for 2OG, suggesting a higher affinity of the R140Q IDH2 variant for **1** than for 2OG ($K_{m}^{app}$ ∼ 2.0 mM; Table 2, entry B). It appears that the R140Q IDH2 $K_{m}^{app}$-value rises on increasing the size of the C3 substituent from methyl to ethyl to propyl, but then drops substantially for the C3 butyl substituent; a similar trend was observed for R132H IDH1, but not for R172K IDH2 (Table 2, entries B-E).

Analysis of $k_{cat}$/$K_{m}$-values from the absorbance assays implies that **2**-**4** and **7** are approximately equally efficient substrates as 2OG for R132H IDH1 ($k_{cat}$/$K_{m}$ ∼ 0.85 mM^−1^•s^−1^; Table 2, entry A); **1** appears to be ∼2-fold more efficient substrate than 2OG ($k_{cat}$/$K_{m}$ ∼ 1.9 mM^−1^•s^−1^; Table 2, entry B). In support of the absorbance assay results, electrochemical studies with R132H IDH1 show that **1** and **12** are more active substrates than 2OG, but with a higher $K_{m}^{app}$ (Fig. S4).

The $k_{cat}$/$K_{m}$-values for R140Q IDH2 imply all the tested 2OG derivatives are more efficient substrates than 2OG ($k_{cat}$/$K_{m}$ ∼ 0.1 mM^−1^•s^−1^; Table 2, entry A), albeit to a varying degrees with **1** and **4** being ∼25-fold more efficient than 2OG ($k_{cat}$/$K_{m}$ ∼ 2.8 and 2.6 mM^−1^•s^−1^ for **1** and **4**, respectively; Table 2). By contrast, based on the $k_{cat}$/$K_{m}$-values, none of the 2OG derivatives were equally or more efficient substrates than 2OG for R172K IDH2. From biocatalytic and inhibition perspectives, the observation that both the conformationally constrained 2OG derivatives **21** and **23** are relatively efficient substrates is of particular interest.

### NMR and electrochemical turnover studies with R132H IDH1

**Figure 3:**
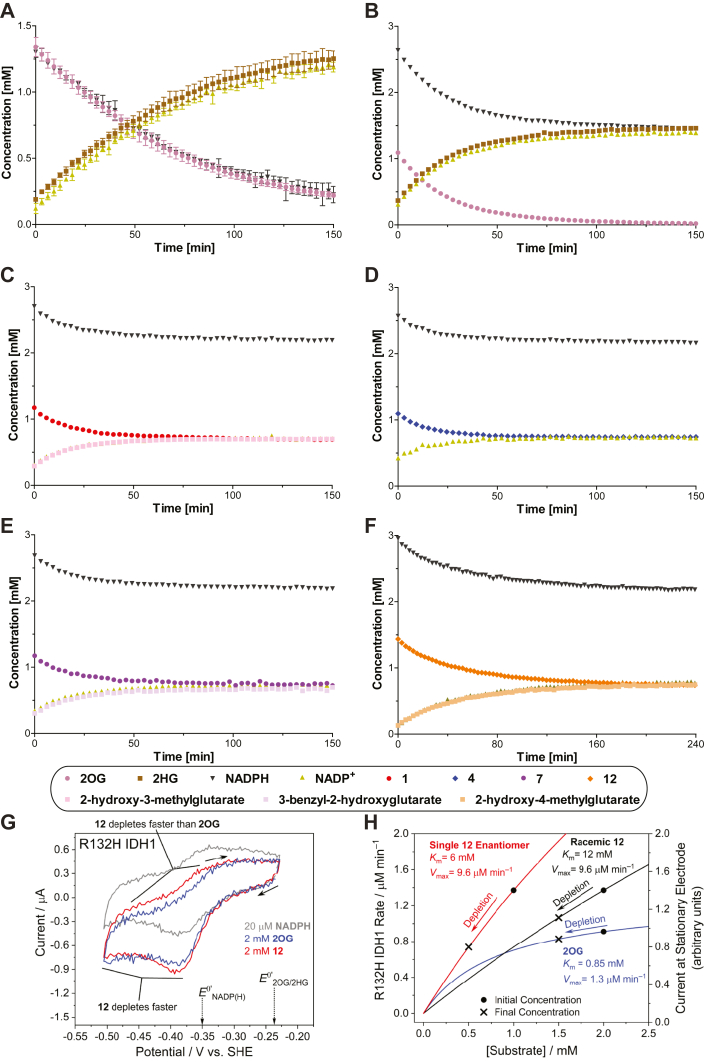
^**1**^**H NMR and electrochemical studies indicate that R132H IDH1 preferentially catalyzes the reduction of one enantiomer from racemic mixtures of C3/C4-substituted 2OG derivatives.**^1^H NMR time course monitoring the R132H IDH1-catalyzed reduction of (*A* and *B*) 2OG, (*C*) **1**, (*D*) **4**, (*E*) **7**, and (*F*) **12** in the presence of equimolar amounts of NADPH (1.5 mM) (*A*) or a twofold excess of NADPH (3.0 mM) and a fourfold greater R132H IDH1 concentration (2.0 μM) (*B*–*F*). The time scales were normalized to the end of the first of 50 subsequent NMR experiments after the addition of R132H IDH1 to the reaction mixture (t = 0 min), by which time low levels of conversion were manifest; (*G*) cyclic voltammograms for a stationary porous electrode containing ‘electroactive’ R132H IDH1 (55) in the presence of 2OG and racemic **12**. The concentration of **12** depletes at the electrode-solution interface faster than 2OG at similar currents (R132H IDH1 faradaic ‘coupled’ current (55) decreased by −43% for **12** compared to −10% for 2OG during each scan); (*H*) Michaelis–Menten curves showing the simulated R132H IDH1 activity based on experimental $K_{m}^{app}$- and $v_{\max}^{app}$-values for 2OG (*blue*), racemic **12** (*black*), and (predicted) enantiopure **12** (*red*). The initial and final concentrations indicate the expected activity/current for each substrate based on the conditions used in panel ***G*** if 500 μM of each substrate was consumed (locally) at the electrode-solution interface during each scan. The predicted changes in catalytic rate (enantiopure **12**: −46%; racemic **12**: −22%; 2OG: −9%) suggest that only one enantiomer of **12** is (at least) an efficient substrate for R132H IDH1. $E_{NADP\left(H\right)}^{0^{\prime }}$ and $E_{2OG/2HG}^{0^{\prime }}$ denote formal potentials for the NADP^+^/NADPH and 2OG/2HG couples, respectively (55). 2OG, 2-oxoglutarate; 2HG, 2-hydroxyglutarate.

R132H IDH1-catalyzed turnover of equimolar mixtures of 2OG and NADPH was monitored by ^1^H NMR; the reduction of representative 2OG derivatives (*i.e.* **1**, **4**, **7**, **12**), substituting for 2OG, was monitored similarly (Fig. S5). However, as the R132H IDH1-catalyzed reductions of 2OG derivatives were slow using an equimolar amount of NADPH, subsequent NMR assays were performed in the presence of a two-fold excess of NADPH with respect to 2OG or the 2OG derivative, a condition which resulted in a substantial increase in turnover over the same time period (Fig. 3). Note, the NMR conditions differ from those of the absorbance and electrochemical assays, which are performed under much more dilute (1, 10) and concentrated (55) conditions, respectively. The results show that the reduction of both 2OG (as anticipated (10)) and of 2OG derivatives is tightly coupled with oxidation of NADPH to NADP^+^ (Fig. 3, *A*–*F*); a single diastereomer was obtained as the alcohol product (Fig. S1). Interestingly, it appeared that for all tested 2OG derivatives (*i.e.* **1**, **4**, **7**, **12**), approximately half of the substrate was only reduced in the presence of excess NADPH (Fig. 3, *C*–*F*). This observation is consistent with the electrochemical results with R132H IDH1 and **12** that imply an effective concentration of **12** which is half that of racemic **12** added (Fig. 3, *G* and *H*). Hence, R132H IDH1-catalyzed reduction of racemic mixtures of C3- or C4-substituted 2OG derivatives appears to be specific for a particular enantiomer.

**Figure 4:**
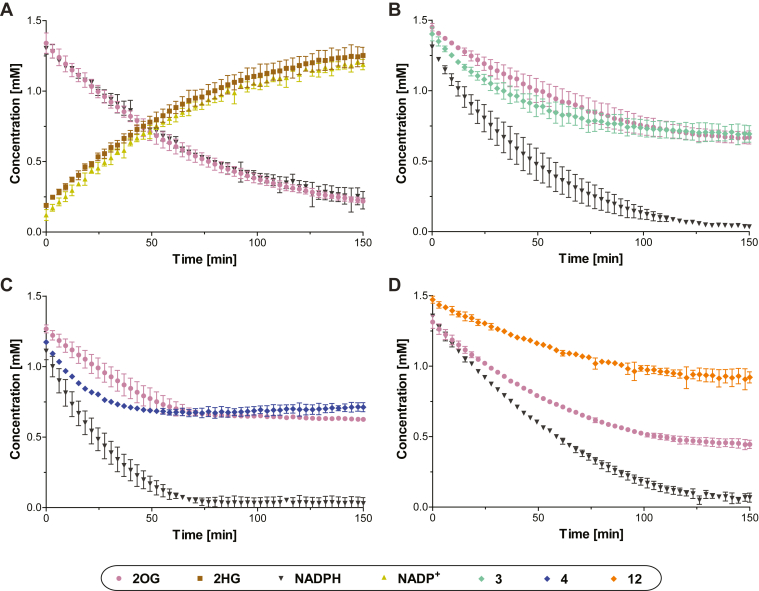
^**1**^**H NMR competition assays provide evidence that 3-butyl–substituted 2OG (4) is a better R132H IDH1 substrate than 2OG under the tested conditions.**^1^H NMR time course monitoring the R132H IDH1-catalyzed reduction of (*A*) 2OG alone (1.5 mM; note that Figure 3*A* and panel (*A*) in this figure are identical, panel (*A*) is shown to help the direct comparison with panels *B*–*D*) and equimolar mixtures of 2OG and (*B*) **3**, (*C*) **4**, or (*D*) **12** in the presence of equimolar NADPH (1.5 mM). The time scales were normalized to the end of the first of 50 subsequent NMR experiments after the addition of R132H IDH1 to the reaction mixture (t = 0 min), by which time low levels of conversion were manifest. Results are a mean of two independent duplicates (n = 2; mean ± SD). 2OG, 2-oxoglutarate.

^1^H NMR assays were employed to monitor the R132H IDH1-catalyzed reduction of 2OG in the presence of an equimolar amount of a 2OG derivative (*i.e.***3**, **4**, **12**) to further inform on potential substrate preferences (such experiments cannot easily be performed employing absorbance assays that monitor NADPH conversion). The results show that, in the presence of 2OG, R132H IDH1 reproducibly catalyzes the reduction of C3 butyl-substituted 2OG derivative **4** and, to a lesser extent, C3 propyl-substituted 2OG derivative **3**, but not the C4 methyl-substituted 2OG derivative **12**, with a preference for the reduction of the 2OG derivative over 2OG (Fig. 4), in accord with the greater $k_{cat}$/$K_{m}$-values of **3** and **4** than those of 2OG (Table 2). Interestingly, the rate of 2OG reduction appeared to be reproducibly lower in the presence of 2OG derivative **3** than in the absence of a 2OG derivative or in the presence of the 2OG derivatives **4** or **12** (Fig. 4). The reasons for these differences are unclear, but they may reflect the reported half-site reactivity of R132H IDH1, which is active as a dimer in solution (1, 54).

### Crystallography

Crystallization trials were initiated to investigate the binding modes of the 2OG derivatives. Structures with R132H IDH1, R172K IDH2, or R140Q IDH2 were not obtained, however, 3-butyl-2OG (**4**) was crystallized in complex with R132C/S280F IDH1 in the presence of catalytically inactive Ca(II), substituting for Mg(II), and NADPH under reported conditions (53). The structure of the R132C/S280F IDH1:Ca(II):**4** complex was solved by molecular replacement using a R132C/S280F IDH1 structure (PDB ID: 7PJM (53)) as a search model (2.35 Å resolution; *C* 2 2 2_1_; Fig. S6 and Table S6).

R132C/S280F IDH1 has been observed in AML patients treated with the R132C IDH1 inhibitors enasidenib or ivosidenib and manifests resistance because it restores IDH1 variant–catalyzed 2HG production (11, 12, 53). The kinetic parameters of R132C/S280F IDH1 for 2OG are in the range of those obtained for 3-methyl-2OG (**1**) and 3-butyl-2OG (**4**), obtained under the same conditions (Table S7). Note that the kinetic parameters of R132C/S280F IDH1 do not substantially differ from those of the R132H IDH1, R172K IDH2 or R140Q IDH2 variants (Table S7).

**Figure 5:**
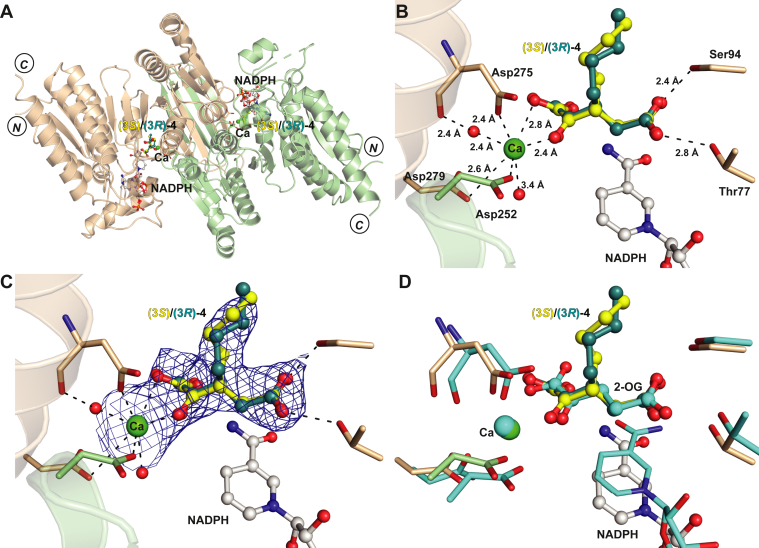
**Crystallographic analysis with R132C/S280F IDH1 reveals the active site–binding mode of 3-butyl-2OG (4).** Color code: R132C/S280F IDH1: monomer 1: wheat, monomer 2: *green*; carbon-backbone of (3*R*)-butyl-2OG [(3*R*)-**4**] in *yellow*, (3*S*)-butyl-2OG [(3*S*)-**4**] in *teal*; NADPH in *gray*; oxygen: *red*; nitrogen: *blue*. *A*, ribbon view of R132C/S280F IDH1:Ca:NADPH:**4** (PDB ID: 8BAY, 2.35 Å resolution) showing the dimer formed by chain A (wheat) and chain B (*green*); *B*, view of the metal and substrate-binding site revealing the Ca(II)-binding residues Aps252 (monomer 2, *green*, helix α9), Asp275 and Asp279 (monomer 1, wheat, α10) and the **4**-binding residues Thr77 and Ser94; (*C*) 2mF_o_-DF_c_ electron density map contoured to 1.0 σ around **4** and Ca(II) in complex with R132C/S280F IDH1 and NADPH; (*D*) superimposition of the active site views from chain A of the R132C/S280F IDH1:Ca:NADPH:**4** structure (PDB ID: 8BAY, 2.35 Å resolution) and the reported R132C/S280F IDH1:Ca(II):NADPH:2OG structure (*cyan*; PDB ID: 7PJM (53)) reveal a similar binding mode and conformation for 2OG and **4**. 2OG, 2-oxoglutarate.

The R132C/S280F IDH1:Ca(II):**4** complex structure reveals that **4** binds at the 2OG-binding site, consistent with the kinetic studies (Fig. 5*A*). Comparison with a reported R132C/S280F IDH1:Ca(II):2OG complex structure (PDB ID: 7PJM (53)) shows that the conformations of the scaffolds of 2OG and **4** are similar (Fig. 5*B*). The *n*-butyl group of **4** projects away from the NADPH molecule; thus, **4** likely does not compete with NADPH binding, consistent with the observation that **4** is an efficient substrate for R132C/S280F IDH1. Note, it was not possible to define the absolute configuration at C3 in **4** complexed with R132C/S280F IDH1, due to the resolution of the structure (2.35 Å; note, we refined and deposited the structure with 50% occupancy of each (*3R*)-butyl-2OG [(*3R*)-**4**] and (*3S*)-butyl-2OG [(*3S*)-**4**]; Polder omit maps of the data refined with solely one enantiomer are shown in Fig. S7). However, it is likely that the reduction of **4** (and by implication other 2OG derivatives) proceeds from the same face as the IDH1/2 variant–catalyzed reduction of 2OG to give the (*2R*)-products, considering the similar orientations of 2OG and **4** in the active site (Fig. 5).

### Synthetic 2OG derivatives inhibit IDH1/2 variants

To further investigate the utility of the C3/C4-substituted 2OG derivatives to modulate the activity of the three cancer-associated IDH1/2 variants, their inhibition by 32 2OG derivatives (22) was investigated using the reported absorbance assays (1, 10). The 2OG-competitive IDH1/2 variant inhibitor NOG was used as a positive control (41). Interestingly, the results reveal that, of the 32 tested derivatives, only **11**, which contains a bulky fluorene-derived substituent at its C3 position and which is not an IDH1/2 variant substrate (Table 1), manifests notable inhibition when assayed at an equimolar ratio with 2OG (Table S8). This observation implies that, except for **11**, interpretation of the kinetic data for substrate conversion is not, at least substantially, compromised by inhibition *via* binding of the nonproductive enantiomer of racemic substrates to the IDH variant active site.

**Figure 6:**
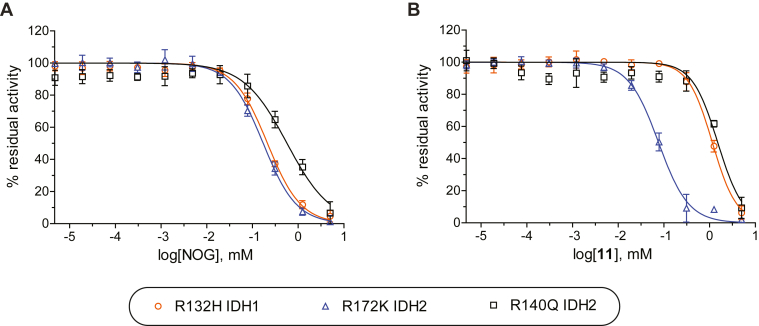
**2OG derivative 11 inhibits R172K IDH2 more efficiently than R132H IDH1 and R140Q IDH2.** Representative dose-response curves used to determine IC_50_ values for (*A*) NOG and (*B*) **11** for R132H IDH1 (*orange circles*), R172K IDH2 (*blue triangles*), and R140Q IDH2 (*black boxes*). Hill coefficients (76) of the inhibition curves were in the range of the expected value of −1 for substrate-competitive IDH1/2 variant inhibitors. 2OG, 2-oxoglutarate; NOG, N-oxalylglycine.

Half maximum inhibitory concentrations (IC_50_ values) for the IDH1/2 variants were determined for **11** and NOG (Table 3 and Fig. 6). The NOG IC_50_ value for R140Q IDH2 (IC_50_ ∼ 621 μM) was three-fold higher than for R132H IDH1 (IC_50_ ∼ 188 μM) and R172K IDH2 (IC_50_ ∼ 161 μM; Table 3, entry A). This observation likely, at least in part, reflects the different 2OG concentrations employed; the 2OG concentration in the R172K IDH2 inhibition assay was around the physiological concentration in healthy cells (∼1 mM (57, 58)), which is lower than the 2OG concentration in the R132H IDH1 (1.5 mM) and R140Q IDH2 (6.0 mM) inhibition assays; note that different 2OG concentrations were used to account for the observed differences in the $K_{m}^{app}$-values (Table 2, entry A).

**Table 3:** Racemic 2OG derivative **11** is a more efficient R172K IDH2 inhibitor than *N*-oxalylglycine

Entry	^a^2OG derivative	IDH1/2 variant	^b^IC_50_ [μM]
A		^c^R132H IDH1	188 ± 26
		^d^R172K IDH2	161 ± 25
		^e^R140Q IDH2	621 ± 69
B		^c^R132H IDH1	1104 ± 62
		^d^R172K IDH2	84 ± 14
		^e^R140Q IDH2	1652 ± 206

^a^ 2OG derivative **11** was prepared as a racemic mixture according to a reported procedure (22).

^b^ determined using absorbance assays as described in the Experimental procedures section, mean of two independent runs each composed of technical quadruplicates (n = 2; mean ± SD).

^c-e^ using 0.03 μM IDH1/2 variant, 50 μM NADPH, and (c) 1.5 mM 2OG, (d) 1.0 mM 2OG, or (e) 6.0 mM 2OG in reaction buffer (100 mM Tris, pH 8.0, 0.005%_v/v_ tween-20, 0.1 mg/mL BSA, 0.2 mM DTT, 10 mM MgCl_2_, 150 mM NaCl).

By contrast with NOG, **11** is more than 10- or 20-fold more efficient in inhibiting R172K IDH2 (IC_50_ ∼ 84 μM) than R132H IDH1 (IC_50_ ∼ 1104 μM) or R140Q IDH2 (IC_50_ ∼ 1652 μM), respectively (Table 3). 2OG derivative **11** is also about two-fold more efficient in inhibiting R172K IDH2 than NOG (IC_50_ ∼ 84 μM for **11** and ∼ 161 μM for NOG; Table 3); it is predicted that one of the enantiomers of **11** will be even more potent. Thus, although **11** is a substantially less efficient IDH1/2 variant inhibitor than the clinically used allosterically binding inhibitors such as ivosidenib (8) and enasidenib (9), the results highlight the potential of 2-oxoacids as active site–binding IDH1/2 variant inhibitors, possibly in an isoform-specific manner. Note that the synthetic 2OG derivatives, including **11**, did not substantially inhibit the activity of WT IDH1/2 in the presence of an equimolar amount of DL-isocitrate (Table S9).

## Discussion

The combined absorbance, ^1^H NMR, and electrochemical assay results reveal that some C3- and C4-substituted 2OG derivatives (*e.g.* 3-butyl-2OG, **4**) are efficient substrates of cancer-associated IDH1/2 variants, *i.e.* R132H IDH1, R132C/S280F IDH1, R172K IDH2, and R140Q IDH2, affording the corresponding alcohol products (Figs. 2 and S1), in accord with spectroscopic and crystallographic evidence showing their binding at the active site (Figs. 5 and S3). Remarkably, several of the 2OG derivatives with IDH1/2 substrate activity are human metabolites (*e.g.* DL-4-hydroxyl-2OG (43, 44)) and/or natural products (*e.g.* DL-4-hydroxy-4-methyl-2OG, parapyruvate (45, 46)), some of which are present in human nutrition, *i.e.* 3-methyl-2OG (**1**) (36) and 4-methyl-2OG (**12**) (37, 38, 39) (Tables 1 and S2).

Kinetic studies indicate that 3-methyl-2OG (**1**) and other 2OG derivatives are equally efficient, or even more efficient substrates of isolated recombinant IDH1/2 variants than 2OG, at least under the tested conditions (Table 2). Note that the interpretation of the kinetic values obtained for racemic mixtures of chiral 2OG derivatives is potentially complicated. However, with the exception of 2OG derivative **11**, none of the tested compounds were potent inhibitors of the tested cancer-associated IDH variants (Table S8). In addition, the NMR studies indicate, at least preferential, conversion of one enantiomer (Fig. 3) and formation of one diastereomer (Fig. S1). Thus, the NMR studies are consistent with the proposal that only one enantiomer binds to the R132H IDH1 active site (Fig. S3).

The combined kinetic results suggest that the substrate scope of cancer-associated IDH1/2 variants in cells may extend beyond 2OG, a proposal which requires further investigation *via* cell-based studies. In this regard, it is notable that (*3S*)-hydroxy-2-oxobutanoic acid, the transamination product of threonine, has recently been proposed as a substrate of IDH variants based on metabolomic studies (59). It will also be of interest to explore whether the reduced 2OG derivatives have a similar effect on cancer progression as identified for 2HG itself (4, 25).

Analysis of the $k_{cat}$/$K_{m}$-values reveals that the substrate preference of the oncogenic IDH1/2 variants depends, at least to some extent, on the nature and location of the mutated residue within the IDH active site, *i.e.* none of the tested 2OG derivatives were more efficient substrates than 2OG for R172K IDH2, while multiple 2OG derivatives, including cyclic **21** and **23**, were more efficient substrates than 2OG for R140Q IDH2 (Table 2). The overall observations are in general accord with previous crystallographic and mass spectrometric evidence on the reactivities of the isolated recombinant human 2OG oxygenases FIH and AspH with 2OG derivatives, which reveal that 2OG derivatives can selectively react with FIH or AspH, an observation likely reflecting differences in the 2OG-binding pockets of FIH and AspH (22, 23). The apparent *in vitro* promiscuity of isolated oncogenic IDH1/2 variants with respect to substrate selectivity and of isolated human 2OG oxygenases with respect to their 2-oxoacid (co)substrates is precedented by the reported substrate promiscuity of, for example, alcohol dehydrogenases (60, 61, 62) and lactate dehydrogenases (63, 64, 65).

2OG is present in high concentrations in healthy cells (∼1 mM (57, 58)), hence, it is expected to be the normally preferred substrate for IDH variants and 2OG oxygenases. It is, however, possible that the 2-oxoacid substrate preference of IDH variants (and other 2OG using enzymes) varies in a manner dependent on the localized availability of 2OG/2OG derivatives, both of which could be affected by nutrition and, at least in the case of 2OG, by multiple metabolic pathways, some of which may be altered in disease, as it is the case for IDH variant bearing cancer cells (4, 13, 14, 15). Thus, at least in some circumstances, there may be competition in cells between 2OG and 2OG derivatives for binding to IDH variant active sites.

The evidence for promiscuity with respect to substrate selectivity of the IDH variants contrasts with the results for inhibition, which reveal that, of the tested 2OG derivatives, only **11** manifests substantial inhibition (Table S8). The ability of 2OG derivative **11**, which is not a substrate for the oncogenic IDH1/2 variants under the tested conditions (Table 1), to inhibit oncogenic IDH1/2 variants supports the proposal that 2OG competitive inhibitors other than NOG could be developed for the selective inhibition of oncogenic IDH1/2 variants (Table 3). 2-Oxoacid–based inhibitors of cancer-associated IDH1/2 variants may complement currently used allosteric inhibitors whose efficacy can be reduced in patients because of inducing treatment-resistant second site IDH mutations. Work on 2OG oxygenase inhibitor development has shown that the 2-oxoacid functional group can be replaced with more pharmacologically preferred groups (42).

It should be noted that 2OG derivative **11** is also a potent inhibitor of the 2OG oxygenase FIH which may limit its utility as a chemical probe and for the development of clinical applications (23). Previously, we have shown that 4,4-dimethyl-2OG (**13**) is an efficient inhibitor of the 2OG oxygenase AspH and does not inhibit functionally related FIH (22, 23). It is also reported that **13** is a less efficient substrate of glutamic oxaloacetic aminotransferase than 2OG (66). The observation that **13** is also not an efficient substrate of oncogenic IDH1/2 variants implies that the 2OG-binding sites of functionally related and unrelated enzymes likely differ sufficiently to enable the development of selective 2-oxoacid–based inhibitors for 2OG oxygenases and, in principle, oncogenic IDH1/2 variants (Table 1, entry N).

The observation that oncogenic IDH1/2 variants accept substrates other than 2OG is also of interest from a biocatalytic perspective because chiral 2-hydroxyacids are used as building blocks in organic synthesis. Dehydrogenases other than oncogenic IDH1/2 variants have been employed in organic synthesis (60, 61, 62, 63, 64, 65, 67), and their reactions can be scaled up for industrial applications (68, 69). Because of their broad substrate scope (Table 1) and apparently high levels of diastereoselectivity and, likely, enantioselectivity (Fig. 3), IDH1/2 variants show that they have considerable potential as biocatalysts for production of chiral alcohols, as demonstrated by the reduction of 3-methyl-2OG, 4-methyl-2OG, and related 2OG derivatives to the corresponding alcohols as a single diastereomer (Figs. S1 and 4). In this regard, the ability of IDH variants, in particular R140Q IDH2, to reduce cyclic 2OG derivatives, such as **21** and **23**, is particularly noteworthy (Table 2).

Although the exact stereochemical identities of the alcohol products are currently unclear, IDH1/2 variants could be developed to catalyze the reduction of specific 2-oxoacids to the corresponding enantiopure 2-hydroxyacids which have potential to act as precursors of biomedically relevant compounds, including as precursors for ring forming reactions, for example, to give lactones, which are present in many natural products and some pharmaceuticals (70). The ability of, at least some, cells to tolerate high levels of IDH1/2 variant–catalyzed 2HG production suggests that it should be possible to achieve useful industrial production of 2-hydroxyacids.

## Experimental procedures

### Synthesis

Naturally occurring 2-oxoacids and structurally related small molecules including TCA cycle intermediates were commercially sourced and used as received. 2OG derivatives were synthesized as racemic mixtures according to reported procedures using cyanosulfur ylide intermediates (22). Stock solutions of all compounds were prepared in deionized water (Milli-Q grade).

### Recombinant enzyme preparation

Recombinant ferredoxin NADP^+^-reductase (FNR) from *Chlamydomonas reinhardtii* was expressed and purified as previously described (53, 55).

Recombinant homodimeric WT IDH1 and IDH1 R132H were produced using *Escherichia coli* BL21(DE3) pLyS cells as described (1); recombinant R132C/S280F IDH1 was prepared as described (53). Recombinant homodimeric wt IDH2, R140Q IDH2, and R172K IDH2 were produced in Sf21 insect cells; the cell pellets were prepared at Evotec. In general, cell pellets from the 10 L Sf21 insect cell cultures were resuspended in 300 mL of lysis buffer (25 mM Hepes, pH 7.5, 200 mM NaCl, 5%_v/v_ glycerol, 1 tablet EDTA-free protease inhibitor per 50 mL lysis buffer; Roche). The mixture was homogenized and cells were lysed by sonication (40% amplitude, 150 s of sonication time, 30 s on/off cycles). The lysates were centrifuged (60 min at 45,000 *g* at 4 °C) and the supernatant lysate was first purified using a HisTrap crude FF 5 mL column (wash buffer: 25 mM Hepes, pH 7.5, 200 mM NaCl, 5%_v/v_ glycerol; elution buffer: 25 mM Hepes, pH 7.5, 200 mM NaCl, 5%_v/v_ glycerol, 500 mM imidazole). After loading the protein onto the column, the column was washed with 15 mL of wash buffer; proteins were then eluted with 4%_v/v_ of elution buffer in wash buffer (20 mM imidazole) over 10 column volumes (CV), followed by a 4 to 100%_v/v_ elution buffer (20–500 mM imidazole) gradient over 10 CV and 100% elution buffer over 5 CV at a flow rate of 4 mL/min. Fractions containing purified protein were combined and concentrated using Amicon Ultra centrifugal filter units (molecular weight cut-off: 30 kDa, Millipore), then further purified by size-exclusion chromatography using a Superdex 200 26/60 column (25 mM Hepes, pH 7.5, 100 mM NaCl, 1 mM DTT). Fractions with purified IDH2 protein were pooled, concentrated, aliquoted, and stored at −80 °C.

### Absorbance assays

The reactivity of WT IDH1/2 and oncogenic IDH1/2 variants was assayed at ambient temperature by continuously monitoring the concentration-dependent change in absorbance (340 nM) of NADPH using a PheraStar FS or a CLARIOstar Plus plate reader (BMG Labtech). Enzyme reactions (60 μL total reaction volume) were performed in clear 384-well UV-STAR plates (Greiner Bio-one, #781801). Compounds were dispensed using a CyBio SDW-CW CyBi-Well Simultaneous Pipettor and assay started using a ThermoFisher Multidrop Combi reagent dispenser.

IDH1/2 activities in the presence of small molecules were measured in reaction buffer (100 mM Tris, pH 8.0, 0.005%_v/v_ tween-20, 0.1 mg/mL BSA, 0.2 mM DTT, 10 mM MgCl_2_, 150 mM NaCl). For wt IDH1/2 assays, final concentrations were as follows: 2 nM IDH1/2 and 75 μM NADP^+^. For IDH1/2 variant assays, final concentrations were as follows: 30 nM IDH1/2 variant and 50 μM NADPH. The concentrations of isocitrate and 2OG are given in the corresponding figure and table legends. Reactions were performed as technical quadruplicates.

The half-maximum inhibitory concentrations (IC_50_) of small molecules for IDH1/2 variants were measured in reaction buffer (100 mM Tris, pH 8.0, 0.005%_v/v_ tween-20, 0.1 mg/mL BSA, 0.2 mM DTT, 10 mM MgCl_2_, 150 mM NaCl). Final assay concentrations were as follows: 30 nM IDH1/2 variant and 50 μM NADPH. The 2OG concentration was varied according to the IDH1/2 variant used: 1.5 mM 2OG for R132H IDH1, 1.0 mM 2OG for R172K IDH2, and 6.0 mM 2OG for R140Q IDH2. Reactions were performed as technical quadruplicates, and all assays were carried out in two independent duplicates. GraphPad Prism (version 5.04) was used for data analysis and to determine the IC_50_ values.

Kinetic parameters of IDH1/2 variants for 2OG derivatives were determined using 30 nM IDH1/2 variant and 50 μM NADPH in reaction buffer (100 mM Tris, pH 8.0, 0.005%_v/v_ tween-20, 0.1 mg/mL BSA, 0.2 mM DTT, 20 mM MgCl_2_, 150 mM NaCl). Reactions were performed as technical duplicates, and all assays were carried out in two independent duplicates. GraphPad Prism (version 5.04) was used for data analysis and to fit data to Michaelis–Menten kinetics.

### ^1^H NMR R132H IDH1 assays

^1^H NMR assays were conducted using a Bruker AVIII 700 MHz NMR spectrometer equipped with a 5-mm inverse triple-resonance-inverse (TCI) cryoprobe at 298 K. NMR spectra were processed using MestReNova (version 1.10) and TopSpin (version 3.6.1). R132H IDH1 assays were performed in buffer (50 mM Tris-*d*_*11*_, pH 7.5, 10%_v/v_ D_2_O, 10 mM MgCl_2_, 150 mM NaCl) using a water suppression pulse sequence (32 scans with a 2 s relaxation delay per time point). Conditions of the R132H IDH1-catalyzed reduction of 2OG derivatives (180 μL total reaction volume in 3 mm diameter MATCH NMR tubes): 0.5 μM R132H IDH1, 1.5 mM 2OG/2OG derivatives, 1.5 mM NADPH.

Binding studies were performed at 298K in buffer (50 mM Tris-*d*_*11*_, pH 7.5, 10%_v/v_ D_2_O, 10 mM CaCl_2_) using CPMG NMR spectroscopy (56) and a water suppression pulse sequence. Concentrated R132H IDH1 (1.4 mM stock) was titrated (in 2.86 μL increments) into a mixture (160 μL total volume) containing a 2OG derivative (50 μM) in 3 mm diameter MATCH NMR tubes (Bruker).

### Electrochemical assays

Assays were carried out in an anaerobic glovebox (Glove Box Technology) under a nitrogen atmosphere. A potentiostat (Autolab PGSTAT 10) running on Nova software was used for all experiments. As previously described, the electrochemical apparatus included an Autolab electrode rotator, a two-chamber custom-made glass electrochemical cell, and custom-made rotating pyrolytic graphite edge (PGE) electrodes (55). Platinum wire was used as the counter electrode; it was placed in the working electrode chamber. Electrode potentials (*E*) were measured against a saturated calomel electrode and converted to the standard hydrogen electrode using a temperature-dependent equation (at 25 °C: *E*_SHE_ = *E*_SCE_ + 0.2412 V) (55). Nanoporous indium tin oxide (ITO) electrodes were made by electrophoretically depositing ITO nanoparticles (<50 nm, Sigma-Aldrich) onto PGE electrodes (ITO/PGE) as previously described (55). Enzymes were loaded into the electrode nanopores by placing 4 to 6 μL of a mixed enzyme solution onto the ITO electrode and allowing it to incubate at ambient temperature for >30 min while ensuring the solution did not evaporate (55). In all experiments, 0.85 nmol (homodimer basis) of IDH1 R132H was used; the amount of coloaded FNR was adjusted to achieve the desired enzyme loading ratio. Electrodes loaded with enzyme were thoroughly rinsed using buffer solution before each experiment to remove excess enzyme. The reaction conditions for the experiment in Figure 3*G* were as follows: Stationary 0.06 cm^2^ (FNR+R132H IDH1)@ITO/PGE electrode, scan rate 2 mV/s, 25 °C, buffer (100 mM Hepes, 150 mM NaCl, pH 8), 10 mM MgCl_2_, 20 μM NADPH, 2.5 mL cell volume, enzyme loading ratio (molar): 1/2 FNR/R132H IDH1 (dimer basis). Conditions for experiments presented in the Supporting Information are given in the figure captions.

### Crystallization and X-ray structure determinations

R132C/S280F IDH1 (25 mg/ml in 20 mM Tris, 100 mM NaCl, pH 7.4) was crystallized as described (53), using the sitting drop vapor diffusion method employing a 24 well Cryschem Plate (Hampton Research) with a reservoir solution of 250 μL and 4 μL drop size. Crystallization conditions were optimized by varying the PEG concentration (PEG3350 15–20%, horizontal axis in steps of 1%) and the salt concentration (calcium acetate 200 or 225 mM, vertical axis), with a constant concentration of Bis-Tris (0.1 M, pH 7) over the entire plate. R132C/S280F IDH1 (25 μL) was incubated for 1 h on ice after addition of 10 mM NADPH (in H_2_O, 10 μL), 20 mM CaCl_2_ (in H_2_O, 5 μL), and 200 mM 3-butyl-2OG **4** (in H_2_O, 10 μL) yielding a final protein concentration of ∼12.5 mg/mL. Crystallization was achieved by the addition of 2 μL of the protein-containing solution to 2 μl precipitant solution employing R132C/S280F IDH1 (53). The plates were sealed with StarSeal Advanced Polyolefin Film (Starlab) and stored at 20 °C with regular monitoring of crystal formation. Crystals (100–200 μM average size) appeared within several days and were selected for data analysis by mixing the crystal-containing droplet in a ratio of 1:1 with reservoir solution containing glycerol (25%_v/v_), followed by harvesting with a nylon loop and cryo-cooling in liquid N_2_.

Data was collected at 100 K using beamline I03 at Diamond Light Source. Data were indexed, integrated, and scaled using the Xia2 (71) strategy of the beamline autoprocessing pipeline (Table S6). The crystal structure was determined by molecular replacement using PHASER (72) employing the R132C/S280F IDH1 complex (PDB: 7PJM (53)) as search model. The structural model was optimized by iterative cycles of manual rebuilding in COOT (73) and crystallographic refinement in Phenix (74) (refinement details are summarized in Table S6).

## Data availability

The crystal structure data for the R132C/S280F IDH1:Ca:NADPH:**4** complex structure is deposited in the protein data bank (PDB) with accession code: 8BAY. Additionally, crystal structure data of the reported R132C/S280F IDH1:Ca(II):NADPH:**2OG** complex structure has been used: 7PJM (53).

## Supporting information

This article contains supporting information (22, 43, 44, 45, 46, 53, 55, 56, 71).

## Conflict of interest

The authors declare that they have no conflict of interest with the contents of this article.
