# Supplementary material for: Natural and synthetic 2-oxoglutarate derivatives are substrates for oncogenic variants of human isocitrate dehydrogenase 1 and 2
Source: J Biol Chem. 2023 Jan 5;299(2):102873. doi: 10.1016/j.jbc.2023.102873 (PMC9939733; doi:10.1016/j.jbc.2023.102873)
Supplement: Supporting information [file mmc1.pdf]

Supporting Information

Natural and Synthetic 2-Oxoglutarate Derivatives are Substrates for Oncogenic Variants of Human Isocitrate Dehydrogenase 1 and 2

Xiao Liu[1], Raphael Reinbold[1], Shuang Liu[1,#], Ryan A. Herold[2,#], Patrick Rabe[1,#], Stéphanie Duclos[3], Rahul B. Yadav[3], Martine I. Abboud[1], Sandrine Thieffine[4], Fraser A. Armstrong[2], Lennart Brewitz[1,*], and Christopher J. Schofield[1,*]

[1]Chemistry Research Laboratory, Department of Chemistry and the Ineos Oxford Institute for Antimicrobial Research, University of Oxford, 12 Mansfield Road, OX1 3TA, Oxford, United Kingdom.

[2]Inorganic Chemistry Laboratory, Department of Chemistry, University of Oxford, South Parks Road, OX1 3QR, Oxford, United Kingdom.

[3]Evotec (UK) Ltd, 90 Park Drive, OX14 4RZ, Abingdon, United Kingdom.

[4]Evotec (US) Inc., 303b College Road East, Princeton, NJ 08540, United States of America.

[#]These authors contributed equally to this work.

*Email: christopher.schofield@chem.ox.ac.uk or lennart.brewitz@chem.ox.ac.uk

Table of contents

1. Supporting figures S2-S11
2. Supporting tables S12-S29
3. References S30

1. Supporting figures

Supporting Figure S1. The oncogenic R132H IDH1 variant catalyzes the reduction of C3/C4 substituted 2OG derivatives to the corresponding C3/C4 substituted 2HG derivatives (continues on the following three pages). NMR and MS analyses of the reaction mixtures of 2OG derivatives treated with R132H IDH1 indicate that R132H IDH1 (and by implication, the other oncogenic IDH1 and IDH2 variants used in this work, *i.e.* R132C/S280F IDH1, R172K IDH2, and R140Q IDH2) catalyzes the reduction of 2OG derivatives to the corresponding alcohol products, similarly to the reported reduction of 2OG to 2HG.

(**a** - **d**) NMR analysis of the reaction mixture of 3-methyl-2OG (**1**) incubated with R132H IDH1. (**a**) Reaction scheme and ^{1}H NMR spectrum of the R132H IDH1 catalyzed reduction of **1**. (**b**) 1D TOCSY NMR spectrum of the assigned reaction product of the R132H IDH1 catalyzed reduction of **1**, *i.e.* 2-hydroxy-3-methylglutarate.

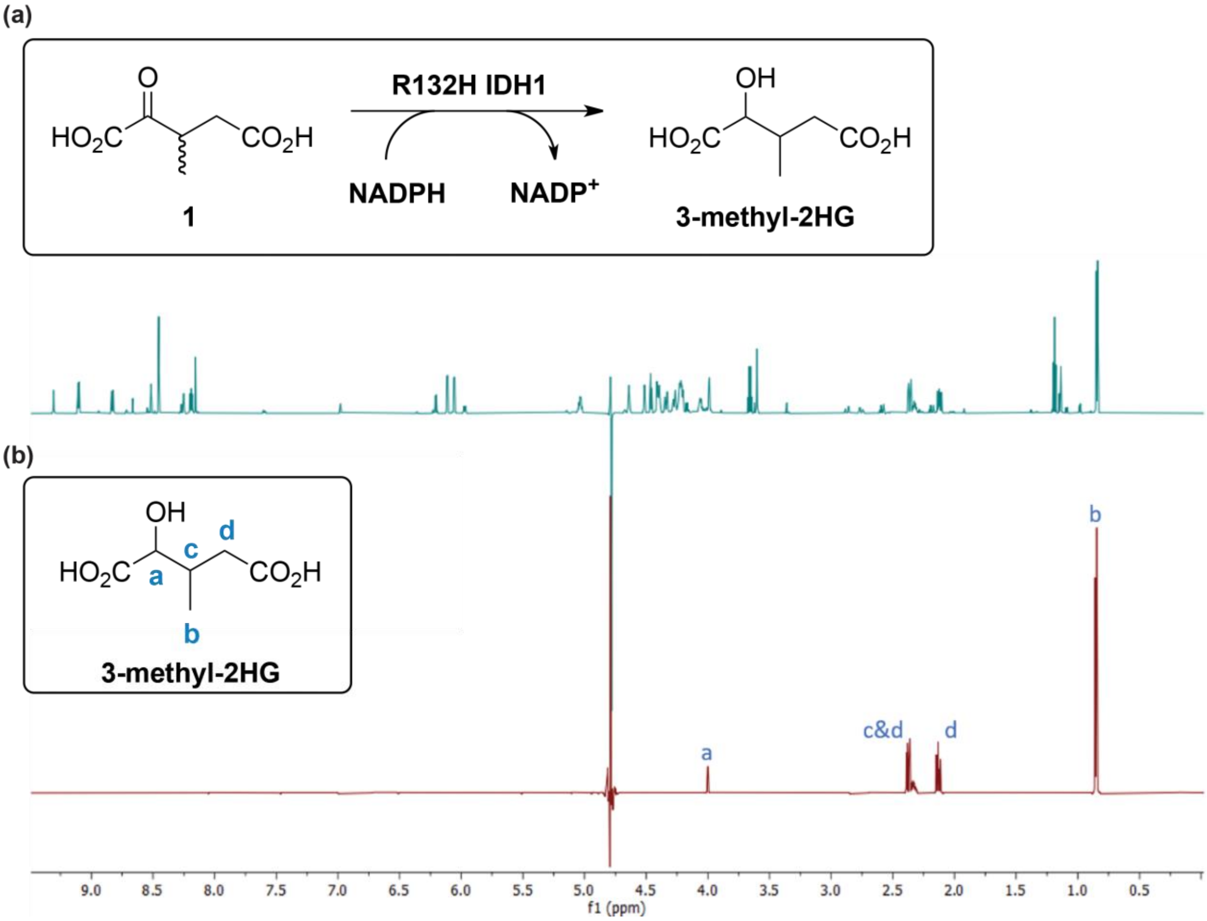

(**c**) HSQC NMR analysis of the reaction mixture of the R132H IDH1-catalyzed reduction of 3-methyl-2OG (**1**) supports the assignment that proton a of the reaction product is connected to a carbon atom which has a chemical shift in the region of that anticipated for C-OH groups. Note that the 1D TOCSY spectrum of the reaction product is shown as the f2 reference.

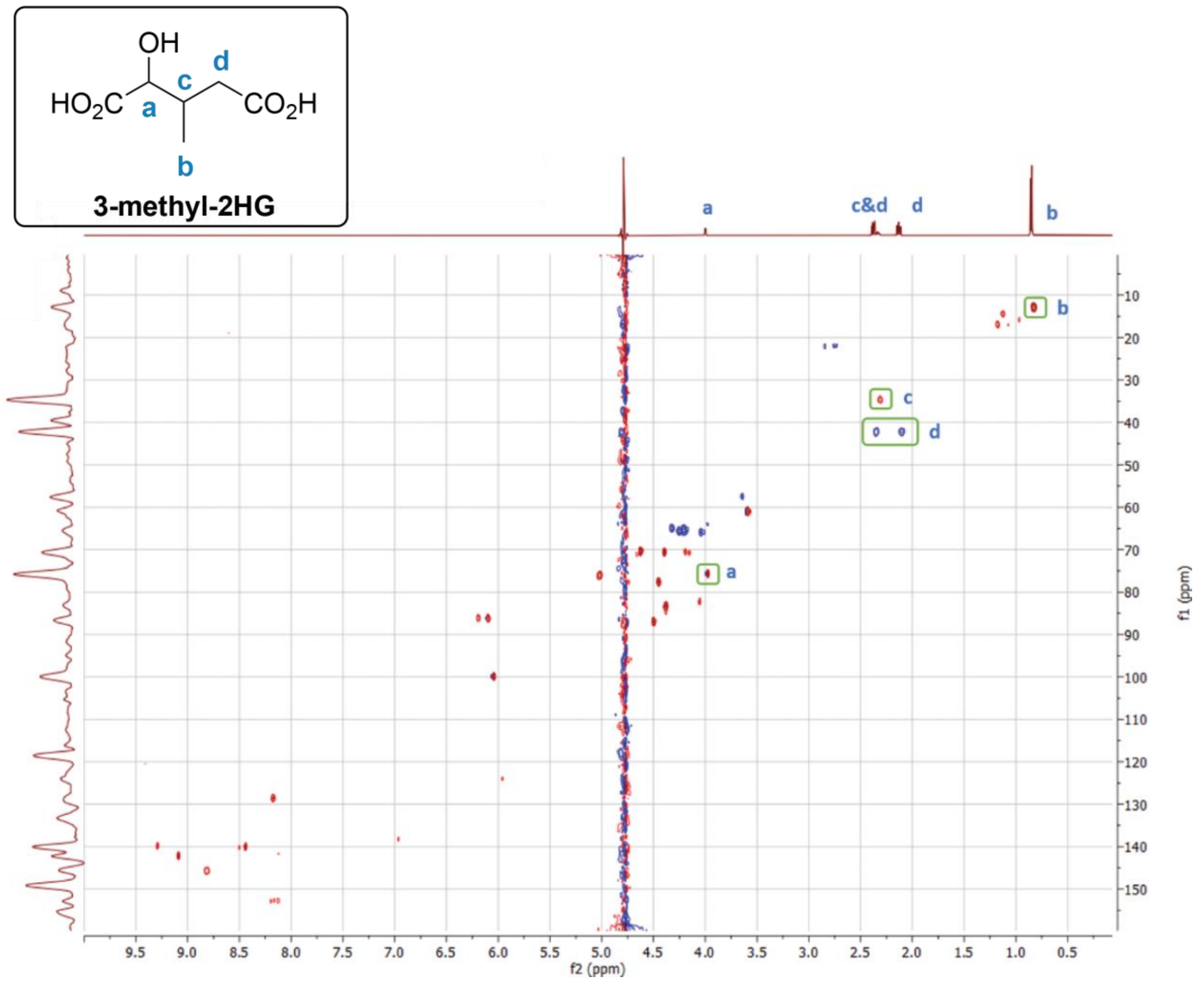

(**d**) HMBC NMR analysis of the reaction mixture of the R132H IDH1-catalyzed reduction of 3-methyl-2OG (**1**) supports the assignment that the reaction product is 2-hydroxy-3-methylglutarate. Note that the 1D TOCSY spectrum of the reaction product is shown as the f2 reference.

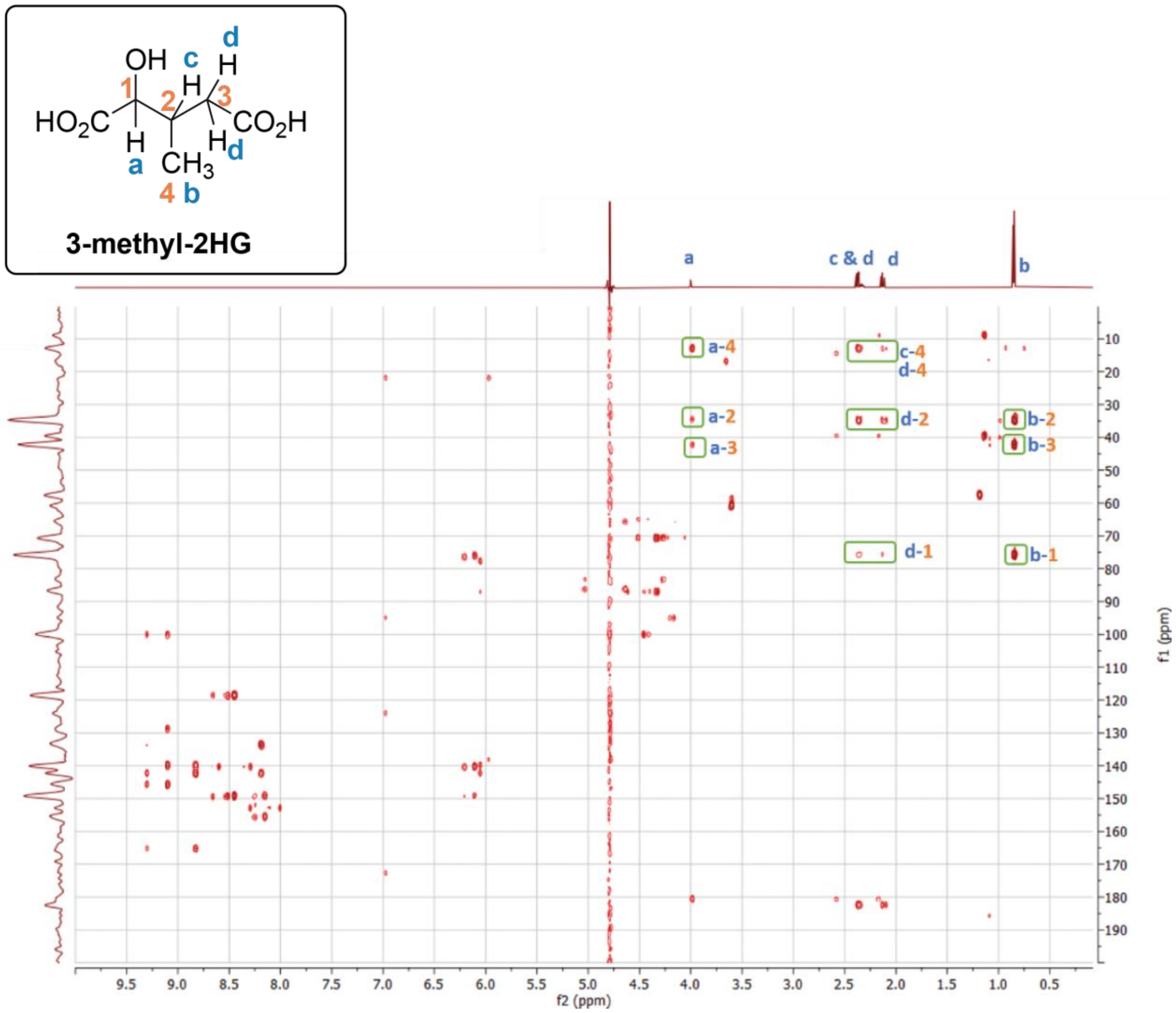

(**e**) Consistent with the NMR analysis of the reaction mixture of the R132H IDH1-catalyzed reduction of 3-methyl-2OG (**1**), MS analysis of the reaction mixture supports the formation of 2-hydroxy-3-methylglutarate (+ ~2 Da mass shift with respect to the ketone starting material; Entry B). Similarly, MS analyses of the reaction mixtures of R132H IDH1 and the 2OG derivatives **4**, **7**, and **21** support the proposal that IDH1/2 variants catalyze the reduction of 2OG derivatives to the corresponding 2HG derivatives.

	[b]**Activity** [%]
A	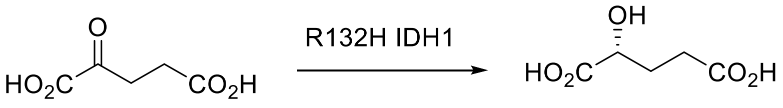<br>**2OG** → **D-2HG**<br>2OG: m/z [M-H]⁻ (AES-MS): 145.0 (calc), 145.0 (found)<br>D-2HG: m/z [M-H]⁻ (AES-MS): 147.0 (calc), 147.0 (found)
B	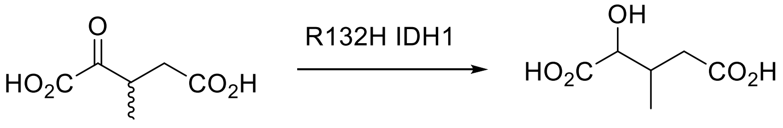<br>**1** → **3-methyl-2HG**<br>1: m/z [M-H]⁻ (AES-MS): 159.0 (calc), 159.0 (found)<br>3-methyl-2HG: m/z [M-H]⁻ (AES-MS): 161.0 (calc), 161.0 (found)
C	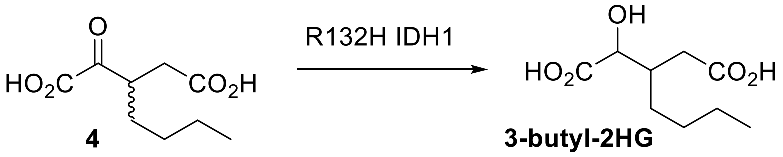<br>**4** → **3-butyl-2HG**<br>4: m/z [M-H]⁻ (AES-MS): 201.1 (calc), 201.1 (found)<br>3-butyl-2HG: m/z [M-H]⁻ (AES-MS): 203.1 (calc), 203.1 (found)
D	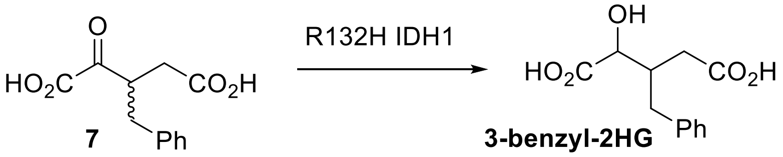<br>**7** → **3-benzyl-2HG**<br>7: m/z [M-H]⁻ (AES-MS): 235.1 (calc), 235.1 (found)<br>3-benzyl-2HG: m/z [M-H]⁻ (AES-MS): 237.1 (calc), 237.1 (found)
E	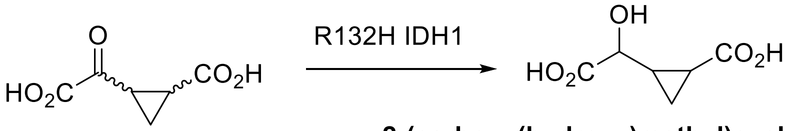<br>**21** → **2-(carboxy(hydroxy)methyl)cyclopropane-1-carboxylic acid**<br>21: m/z [M-H]⁻ (AES-MS): 157.0 (calc), 157.0 (found)<br>2-(carboxy(hydroxy)methyl)cyclopropane-1-carboxylic acid: m/z [M-H]⁻ (AES-MS): 159.0 (calc), 159.0 (found)

Supporting Figure S2. Absorbance-based and electrochemical R132H IDH1 assays afford similar Michaelis constants for 2OG. Michaelis-Menten plot for R132H IDH1 when its activity (rate of 2OG reduction) is measured and controlled electrochemically under steady-state conditions (1). The inlaid figure shows a chronoamperometry experiment where 2OG was titrated into the electrochemical cell solution at increasing concentrations with the resulting measured current being proportional to R132H IDH1 activity (injection arrows show the final concentration of 2OG that was added each time, not the cumulative concentration). The main figure shows the measured current (R132H IDH1 activity) at each 2OG concentration fitted by the Michaelis-Menten equation (K_m ~ 0.6 mM). The results reveal that the Michaelis constant determined using electrochemical assays is similar to that determined using absorbance assays (K_m^{app} ~ 0.85 mM; Table 2, entry A). Conditions: 25 °C, mixed buffer (20 mM each MES, TAPS, CHES, pH 8.0), 10 mM $MgCl_2$, 10 μM NADPH, 4 mL cell volume, (FNR+R132H IDH1)@ITO/PGE electrode, electrode area 0.06 cm^2, rotated at 1000 rpm, E = –0.513 V vs SHE, enzyme loading ratios (molar): FNR/R132H IDH1; 2.5 (dimer basis).

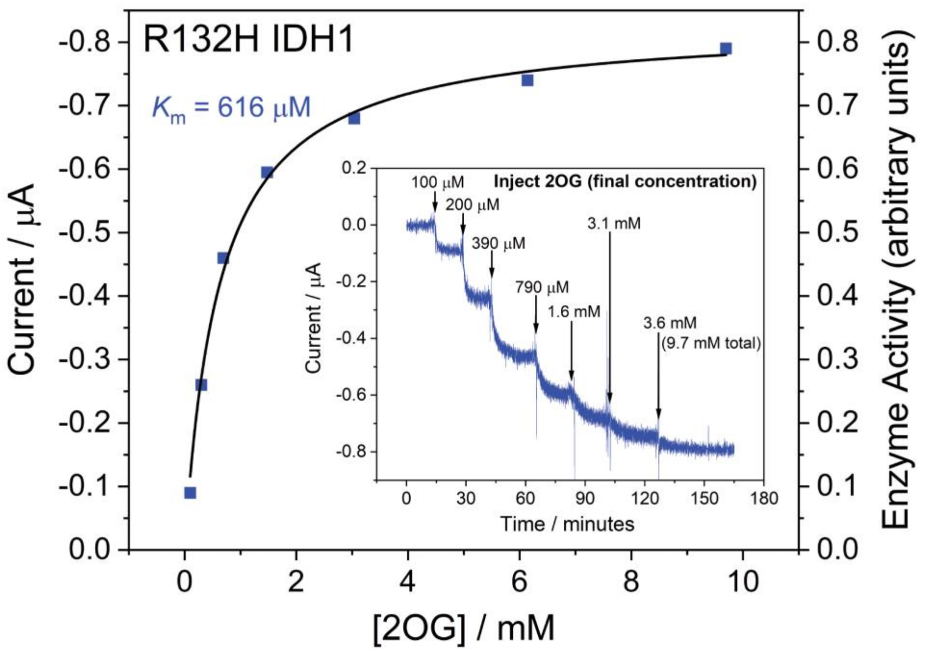

Supporting Figure S3. ¹H NMR binding studies indicate that 2OG derivatives bind to R132H IDH1 in a similar manner as does 2OG. ¹H NMR (CPMG-edited (2), 700 MHz) binding studies of R132H IDH1 with (**a**) 2OG, (**b**) 3-methyl-2OG (**1**), (**c**) 3-butyl-2OG (**4**), (**d**) 3-benzyl-2OG (**7**), and (**e**) 4-methyl-2OG (**12**). * Indicates the position of the protons in the 2OG/2OG derivative corresponding to the displayed peaks. Blue spectra: 50 µM 2OG/2OG derivative. Green spectra: 50 µM 2OG/2OG derivative and 25 µM R132H IDH1. Red spectra: 50 µM 2OG/2OG derivative and 50 µM R132H IDH1. Assays were performed as described in the Experimental Procedures Section by titrating a concentrated R132H IDH1 stock solution (1.4 mM) into a mixture containing 2OG or a 2OG derivative (50 µM) in buffer (50 mM Tris-d_{11}, pH 7.5, 10%$_{v/v}$ D_2O, 10 mM $CaCl_2$).

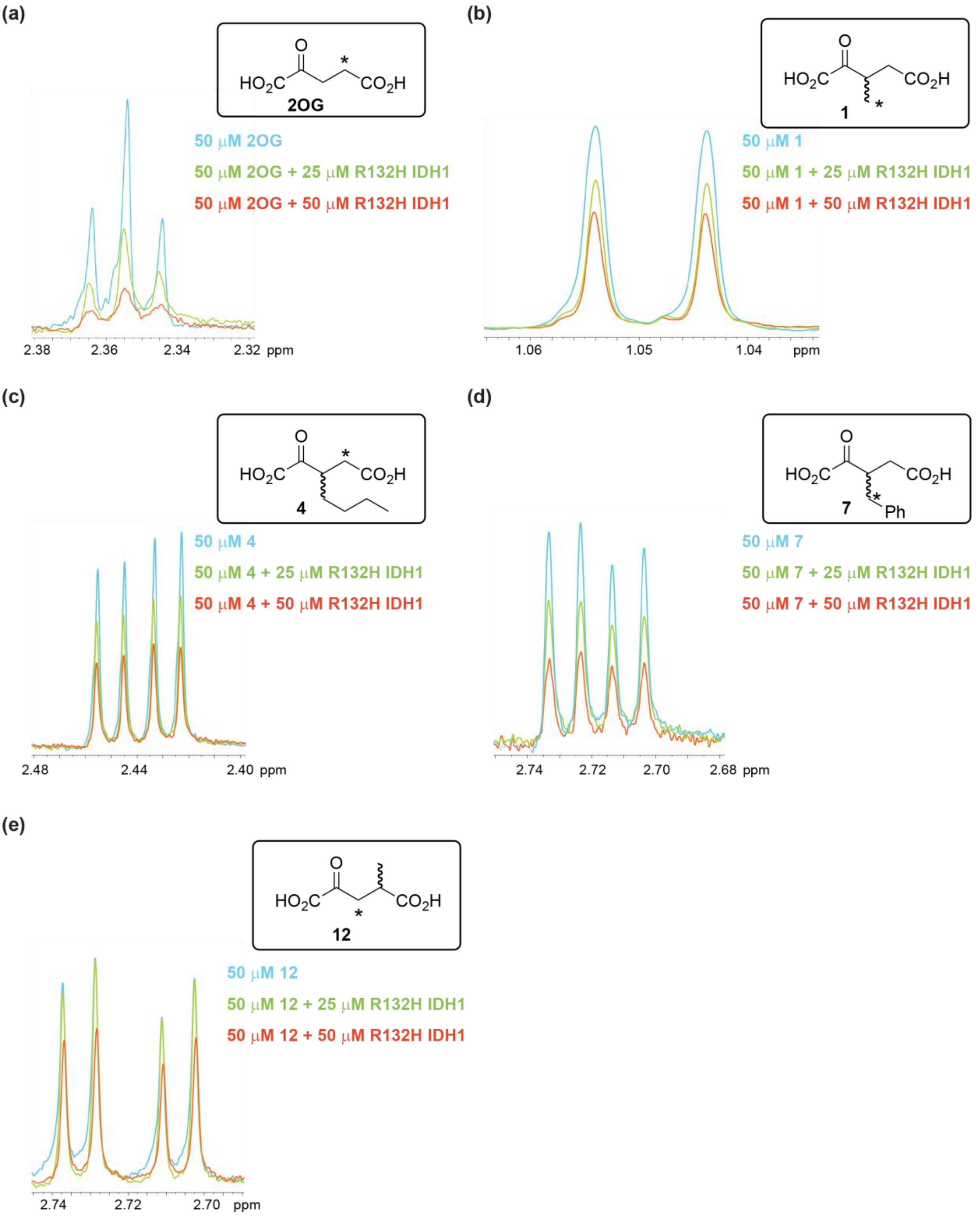

Supporting Figure S4. Electrochemical studies indicate that 3-methyl-2OG (1) and 4-methyl-2OG (12) are more active R132H IDH1 substrates than 2OG but have a lower affinity. Stationary cyclic voltammograms performed at varying concentrations of (**a-c**) 3-methyl-2OG (**1**) and (**d-f**) 4-methyl-2OG (**12**), *i.e.* 2 mM (**a**, **d**; note that Panel **d** is based on the same data plot as Figure 3g, Panel **d** was included to enable the direct comparison with Panels **a**, **e**, and **f**), 4 mM (**b**, **e**), 8 mM (**c**, **f**), indicate that **1** and **12** are more active R132H IDH1 substrates than 2OG at high substrate concentrations. The relative rates of enzyme turnover (represented as faradaic current (1)) are displayed as a function of the applied electrode potential. The grey shaded region in (**a**) shows where 2OG and **1** are reduced to the corresponding 2HG derivative; the applied potential is sufficiently negative to drive reduction and the current is more negative than the background 20 µM NADPH trace (black). For each set of experiments (**a-c** and **d-f**), 2OG was titrated into the electrochemical cell and a scan was performed (to measure activity) at 2 mM, 4 mM, and 8 mM before the reaction buffer was exchanged (removing 2OG). The procedure was repeated for each 2OG derivative (*i.e.*, the same R132H IDH1 sample/electrode was tested against both 2OG and the 2OG derivative under the same conditions to allow direct comparison of enzyme activity at each concentration). Conditions (**a-f**): 25 °C, buffer (100 mM HEPES, 150 mM NaCl, pH 8.0), 10 mM $MgCl_2$, 20 µM NADPH, 2.5 mL cell volume, stationary (FNR+R132H IDH1)@ITO/PGE electrode, enzyme loading ratios (molar): FNR/R132H IDH1; 1/2 (dimer basis). (**a-c**) Electrode area 0.03 cm^2, scan rate 1 mV/s; (**d-f**) electrode area 0.06 cm^2, scan rate 2 mV/s. $E^{0\prime}_{NADP(H)}$ and $E^{0\prime}_{2OG/2HG}$ denote formal potentials for the $NADP^+$/NADPH and 2OG/2HG couples, respectively.

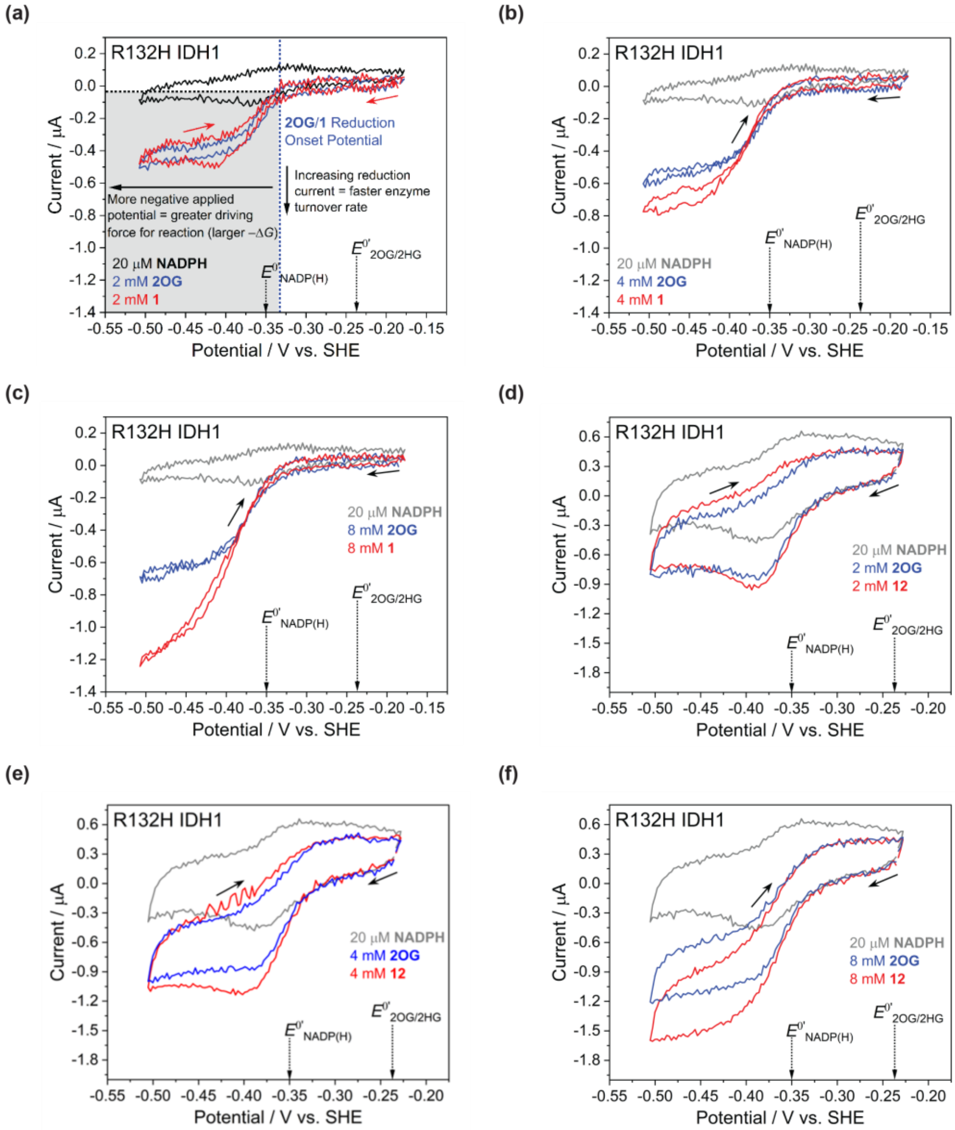

Supporting Figure S5. ¹H NMR studies indicate that the R132H IDH1-catalyzed reduction of 2OG derivatives is slow in the presence of equimolar amounts of 2OG derivative and NADPH. ¹H NMR time course monitoring the R132H IDH1 catalyzed reduction of (**a**) **1**, (**b**) **2**, (**c**) **3**, (**d**) **4**, (**e**) **7**, and (**f**) **12** in the presence of equimolar amounts of NADPH. ¹H NMR assays were performed as described in the Experimental Procedures Section using a Bruker AVIII 700 MHz NMR spectrometer equipped with a 5-mm inverse triple-resonance-inverse (TCI) cryoprobe at 298 K. Conditions: 0.5 μM R132H IDH1, 1.5 mM 2OG derivative, and 1.5 mM NADPH in buffer (50 mM Tris-d_{11}, pH 7.5, 10%$_{v/v}$ D_2O, 10 mM $MgCl_2$, 150 mM NaCl). The time scales were normalized to the end of the first of 50 subsequent NMR experiments after the addition of R132H IDH1 to the reaction mixture (t = 0 min), by which time low levels of conversion were manifest.

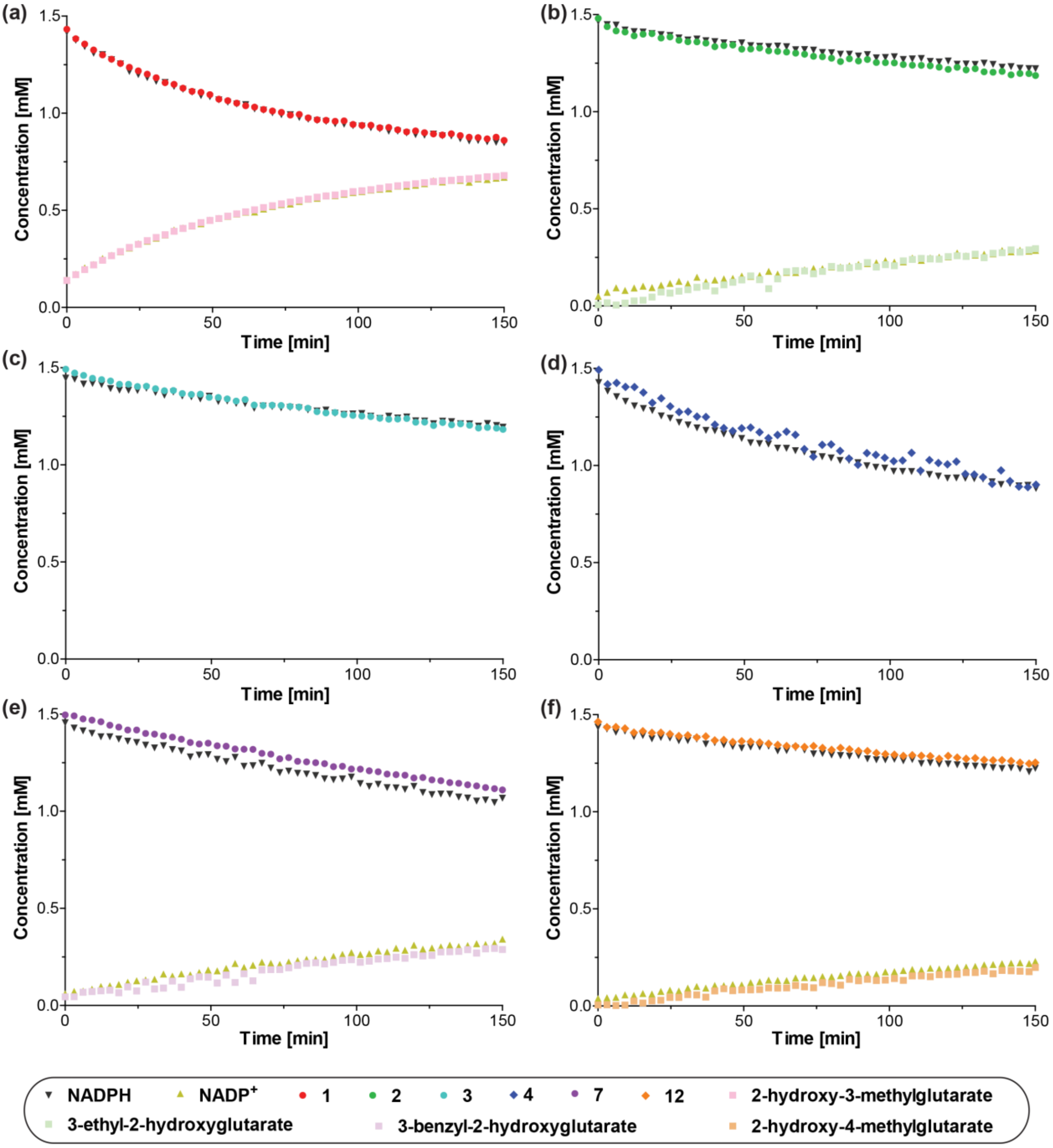

Supporting Figure S6. Crystal structure derived views of R132C/S280F IDH1 in complex with NADPH and 3-butyl-2OG (4). (**a**) Ribbon view of R132C/S280F IDH1 (PDB: 8BAY, 2.35 Å resolution) in complex with Ca, NADPH and 3-butyl-2OG (**4**). 3 R132C/S280F IDH monomers crystallize in the asymmetric unit (chain A (wheat), chain B (green), chain C (yellow)), with chains A and B forming a dimer in the same asymmetric unit; chain C interacts with chain C (gray) from an adjacent asymmetric unit to form a dimer. Carbon-backbone of (3*R*)-3-butyl-2OG [(3*R*)-**4**] is in yellow, (3*S*)-3-butyl-2OG [(3*S*)-**4**] is in teal; NADPH is in light gray; oxygen: red; nitrogen: blue. (**b**) Superimposition of the folds of the R132C/S280F IDH1:Ca:NADPH:**4** complex (PDB ID: 8BAY) and the reported R132C/S280F IDH1:Ca(II):NADPH:2OG complex (cyan; PDB ID: 7PJM (3)) reveal similar conformations, with some deviations in the conformation of chain C (C-α RMSD: 1.03 Å).

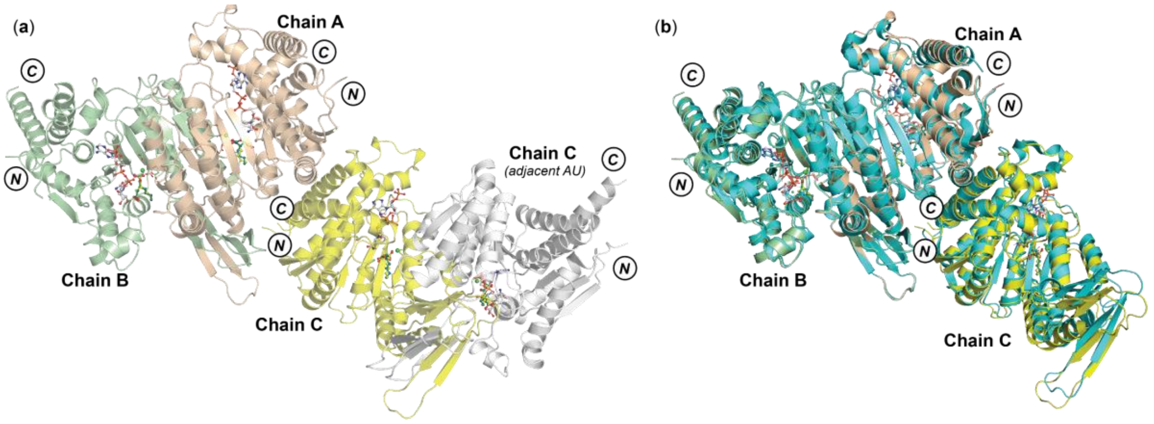

Supporting Figure S7. Active site analysis of R132C/S280F IDH1 in complex with NADPH and butyl-2OG (4). (**a-c**) Polder OMIT electron density map of chain A of the R132C/S280F IDH1:Ca:NADPH:**4** complex contoured to 3.0 σ refined with (**a**) a mixture of (3*R*)-butyl-2OG [(3*R*)-**4**, 50%] in yellow, (3*S*)-butyl-2OG [(3*S*)-**4**, 50%]] in teal, (**b**) solely (3*S*)-butyl-2OG [(3*S*)-**4**, 100%]] in teal and (**c**) solely (3*R*)-butyl-2OG [(3*R*)-**4**, 100%]] in yellow. (**d-f**) Polder OMIT electron density map of chain B of the R132C/S280F IDH1:Ca:NADPH:**4** complex contoured to 3.0 σ refined with (**d**) a mixture of (3*R*)-butyl-2OG [(3*R*)-**4**, 50%] in yellow, (3*S*)-butyl-2OG [(3*S*)-**4**, 50%]] in teal, (**e**) solely (3*S*)-butyl-2OG [(3*S*)-**4**, 100%]] in teal and (**f**) solely (3*R*)-butyl-2OG [(3*R*)-**4**, 100%]] in yellow. (**g-i**) Polder OMIT electron density map of chain C of the R132C/S280F IDH1:Ca:NADPH:**4** complex contoured to 3.0 σ refined with (**g**) a mixture of (3*R*)-butyl-2OG [(3*R*)-**4**, 50%] in yellow, (3*S*)-butyl-2OG [(3*S*)-**4**, 50%]] in teal, (**h**) solely (3*S*)-butyl-2OG [(3*S*)-**4**, 100%]] in teal and (**i**) solely (3*R*)-butyl-2OG [(3*R*)-**4**, 100%]] in yellow. Note: Due to the limited resolution, it was not possible to determine with confidence which enantiomer of **4** binds the IDH active site. Therefore, the structure has been deposited in the PDB with both enantiomers of **4** at a 50% occupancy level.

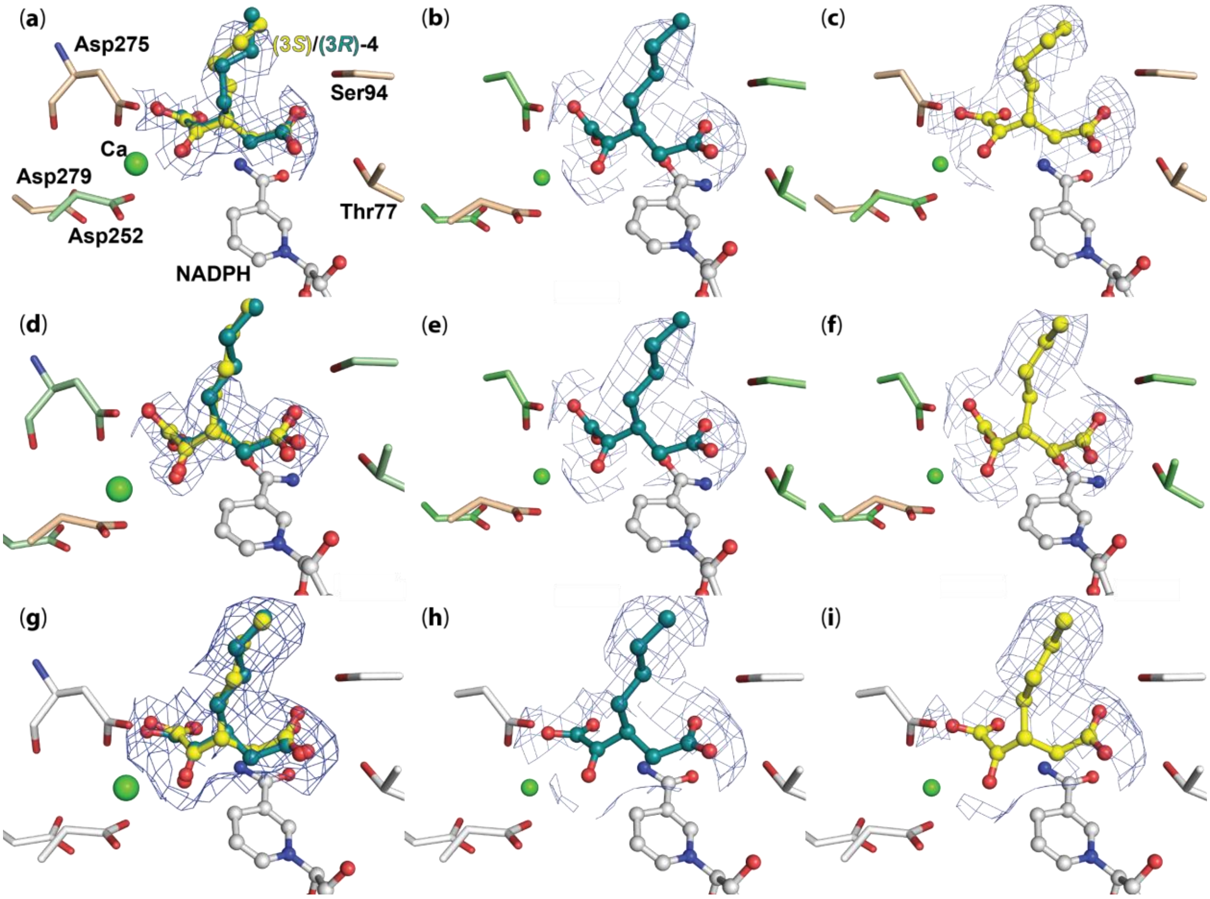

2. Supporting tables

Supporting Table S1. *N*-Oxalylglycine is an efficient inhibitor of oncogenic IDH1/2 variants (continues on the following two pages). The inhibitory effects of 43 2-oxoacids and structurally related small-molecules, including TCA cycle intermediates, on isolated recombinant human R132H IDH1, R172K IDH2, and R140Q IDH2 was investigated in the presence of equimolar amounts of 2OG as a substrate. The results reveal that of the tested 43 small-molecules only *N*-oxalylglycine (NOG; Entry AI) inhibited the activity of the three IDH1/2 variants efficiently (≥90% inhibition). DL-Isocitric acid appeared to inhibit all three tested oncogenic IDH1/2 variants (Entry X), whereas mercaptopyruvic acid inhibited R132H IDH1 and R172K IDH2 (~50% inhibition, Entry F) but not R140Q IDH2.

	[a]**2-oxoacids and related small-molecules**	**IDH1/2 variant**	[b]**activity** [%]		[a]**2-oxoacids and related small-molecules**	**IDH1/2 variant**	[b]**activity** [%]
A	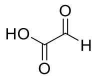 **glyoxylic acid**	[c]R132H	108 ± 5	H	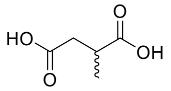 ***DL*-2-methylsuccinic acid**	[c]R132H	104 ± 3
		[d]R172K	99 ± 6			[d]R172K	102 ± 9
		[e]R140Q	105 ± 6			[e]R140Q	115 ± 12
B	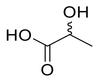 ***DL*-lactic acid**	[c]R132H	104 ± 8	I	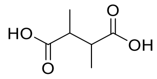 **1,2-dimethylsuccinic acid**	[c]R132H	100 ± 2
		[d]R172K	100 ± 8			[d]R172K	99 ± 5
		[e]R140Q	115 ± 9			[e]R140Q	110 ± 3
C	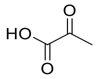 **pyruvic acid**	[c]R132H	112 ± 8	J	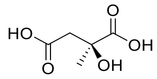 **(*R*)-citramalic acid**	[c]R132H	95 ± 2
		[d]R172K	114 ± 12			[d]R172K	100 ± 5
		[e]R140Q	108 ± 4			[e]R140Q	110 ± 9
D	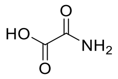 **oxamic acid**	[c]R132H	99 ± 2	K	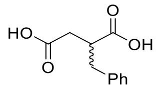 ***DL*-benzylsuccinic acid**	[c]R132H	93 ± 3
		[d]R172K	102 ± 7			[d]R172K	98 ± 3
		[e]R140Q	117 ± 3			[e]R140Q	123 ± 5
E	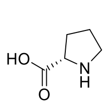 ***L*-proline**	[c]R132H	98 ± 1	L	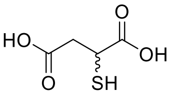 **2-mercaptosuccinic acid**	[c]R132H	104 ± 4
		[d]R172K	98 ± 4			[d]R172K	99 ± 4
		[e]R140Q	106 ± 2			[e]R140Q	91 ± 5
F	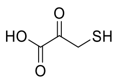 **mercaptopyruvic acid**	[c]R132H	51 ± 5	M	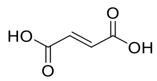 **fumaric acid**	[c]R132H	86 ± 3
		[d]R172K	54 ± 1			[d]R172K	87 ± 6
		[e]R140Q	91 ± 5			[e]R140Q	112 ± 13
G	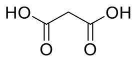 **malonic acid**	[c]R132H	103 ± 4	N	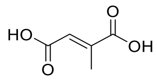 **mesaconic acid**	[c]R132H	105 ± 3
		[d]R172K	98 ± 4			[d]R172K	104 ± 5
		[e]R140Q	117 ± 11			[e]R140Q	116 ± 9

	[a]2-oxoacids and related small-molecules	IDH1/2 variant	[b]activity [%]		[a]2-oxoacids and related small-molecules	IDH1/2 variant	[b]activity [%]
O	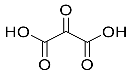 **ketomalonic acid**	[c]R132H	79 ± 1	Y	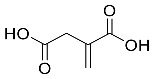 **itaconic acid**	[c]R132H	92 ± 2
		[d]R172K	85 ± 12			[d]R172K	95 ± 7
		[e]R140Q	ND			[e]R140Q	96 ± 3
P	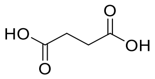 **succinic acid**	[c]R132H	100 ± 4	Z	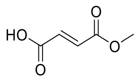 **mono-methyl fumarate**	[c]R132H	90 ± 4
		[d]R172K	89 ± 2			[d]R172K	92 ± 6
		[e]R140Q	124 ± 11			[e]R140Q	98 ± 4
Q	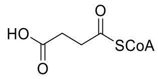 **succinyl CoA**	[c]R132H	73 ± 1	AA	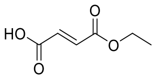 **mono-ethyl fumarate**	[c]R132H	94 ± 8
		[d]R172K	91 ± 6			[d]R172K	107 ± 3
		[e]R140Q	117 ± 2			[e]R140Q	108 ± 7
R	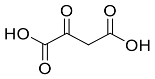 **oxaloacetic acid**	[c]R132H	106 ± 2	AB	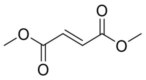 **dimethyl fumarate**	[c]R132H	85 ± 3
		[d]R172K	96 ± 8			[d]R172K	91 ± 2
		[e]R140Q	110 ± 3			[e]R140Q	61 ± 11
S	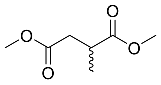 ***DL*-dimethyl 2-methylsuccinate**	[c]R132H	104 ± 2	AC	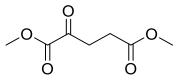 **dimethyl 2-oxoglutaric acid**	[c]R132H	95 ± 3
		[d]R172K	91 ± 4			[d]R172K	100 ± 9
		[e]R140Q	116 ± 4			[e]R140Q	124 ± 12
T	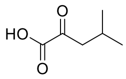 ***α*-ketoisocaproic acid**	[c]R132H	99 ± 2	AD	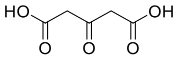 **3-oxoglutaric acid**	[c]R132H	95 ± 2
		[d]R172K	104 ± 2			[d]R172K	100 ± 4
		[e]R140Q	106 ± 6			[e]R140Q	109 ± 3
U	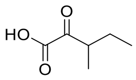 ***α*-ketoisoleucine**	[c]R132H	104 ± 2	AE	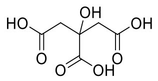 ***DL*-citric acid**	[c]R132H	115 ± 25
		[d]R172K	94 ± 5			[d]R172K	94 ± 4
		[e]R140Q	102 ± 5			[e]R140Q	122 ± 14
V	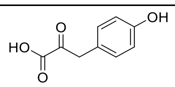 **hydroxyphenylpyruvic acid**	[c]R132H	76 ± 1	AF	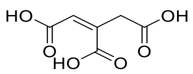 ***cis*-aconitic acid**	[c]R132H	100 ± 2
		[d]R172K	97 ± 6			[d]R172K	99 ± 7
		[e]R140Q	ND			[e]R140Q	119 ± 12
W	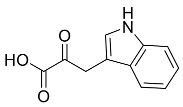 **indole-3-pyruvic acid**	[c]R132H	81 ± 6	AG	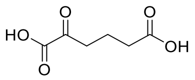 **2-oxoadipic acid**	[c]R132H	115 ± 8
		[d]R172K	87 ± 10			[d]R172K	96 ± 18
		[e]R140Q	ND			[e]R140Q	94 ± 8
X	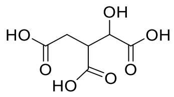 ***DL*-isocitric acid**	[c]R132H	78 ± 5	AH	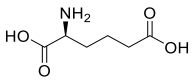 ***L*-2-aminoadipic acid**	[c]R132H	104 ± 7
		[d]R172K	71 ± 5			[d]R172K	104 ± 1
		[e]R140Q	64 ± 3			[e]R140Q	82 ± 4

	[a]2-oxoacids and related small-molecules	IDH1/2 variant	[b]activity [%]
AI	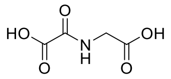 ***N*-oxalylglycine**	[c]R132H	<10
		[d]R172K	13 ± 4
		[e]R140Q	12 ± 7
AJ	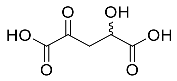 ***DL*-4-hydroxy-2-oxoglutaric acid**	[c]R132H	82 ± 5
		[d]R172K	98 ± 3
		[e]R140Q	124 ± 8
AK	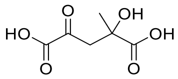 ***DL*-4-hydroxy-4-methyl-2-oxoglutaric acid**	[c]R132H	102 ± 6
		[d]R172K	99 ± 4
		[e]R140Q	116 ± 2
AL	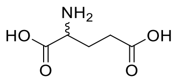 ***DL*-glutamic acid**	[c]R132H	83 ± 5
		[d]R172K	93 ± 5
		[e]R140Q	94 ± 9
AM	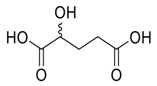 ***DL*-2-hydroglutaric acid**	[c]R132H	89 ± 5
		[d]R172K	95 ± 4
		[e]R140Q	98 ± 8
AN	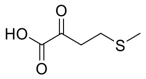 **4-methylthio-2-oxobutyric acid**	[c]R132H	97 ± 1
		[d]R172K	96 ± 1
		[e]R140Q	99 ± 5
AO	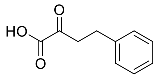 **2-oxo-4-phenylbutanoic acid**	[c]R132H	90 ± 3
		[d]R172K	96 ± 1
		[e]R140Q	98 ± 8
AP	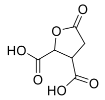 ***DL*-isocitric acid lactone**	[c]R132H	99 ± 2
		[d]R172K	95 ± 3
		[e]R140Q	118 ± 18
AQ	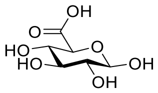 ***D*-glucuronic acid**	[c]R132H	104 ± 4
		[d]R172K	101 ± 2
		[e]R140Q	112 ± 7

a) Small-molecules were employed as metal salts in the assay; b) %-substrate conversion after 20 min incubation with IDH1/2 variants; absorbance assays were performed as described in the Experimental Procedures Section; c-e) using 50 µM NADPH and c) 0.03 µM R132H IDH1 and 1.5 mM 2OG and small-molecule, d) 0.03 µM R172K IDH2 and 1.0 mM 2OG and small-molecule or e) 0.03 µM R140Q IDH2 and 6.0 mM 2OG and small-molecule in reaction buffer (100 mM Tris, pH 8.0, 0.005%$_{v/v}$ tween-20, 0.1 mg/mL BSA, 0.2 mM DTT, 10 mM $MgCl_2$,150 mM NaCl); Results are a mean of four technical replicates (n = 4; mean ± standard deviation, SD). ND: not determined due to intrinsic UV absorbance.

Supporting Table S2. The natural products DL-4-hydroxy-2OG and DL-4-hydroxy-4-methyl-2OG are substrates for the oncogenic R140Q IDH2 variant (continues on the following two pages). The substrate activities of 43 2-oxoacids and structurally related small-molecules, including TCA cycle intermediates, with isolated recombinant human R132H IDH1, R172K IDH2, and R140Q IDH2 was investigated in the absence of 2OG as a substrate. The results reveal that of the tested 43 small-molecules only the human metabolite DL-4-hydroxy-2OG (Entry AJ) (4,5) and the plant and bacterial metabolite DL-4-hydroxy-4-methyl-2OG (parapyruvate, Entry AK) (6,7) were substrates for R140Q IDH2, manifesting ~20% substrate activity compared to 2OG (100% substrate activity).

	[a]**2-oxoacids and related small-molecules**	**IDH1/2 variant**	[b]**activity** [%]		[a]**2-oxoacids and related small-molecules**	**IDH1/2 variant**	[b]**activity** [%]
A	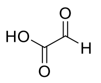 **glyoxylic acid**	[c]R132H	<10	H	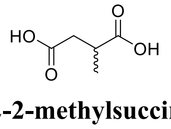 ***DL*-2-methylsuccinic acid**	[c]R132H	<10
		[d]R172K	<10			[d]R172K	<10
		[e]R140Q	<10			[e]R140Q	<10
B	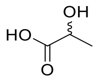 ***DL*-lactic acid**	[c]R132H	<10	I	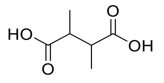 **1,2-dimethylsuccinic acid**	[c]R132H	<10
		[d]R172K	<10			[d]R172K	<10
		[e]R140Q	<10			[e]R140Q	<10
C	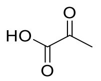 **pyruvic acid**	[c]R132H	<10	J	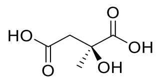 **(*R*)-citramalic acid**	[c]R132H	<10
		[d]R172K	<10			[d]R172K	<10
		[e]R140Q	<10			[e]R140Q	<10
D	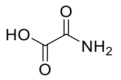 **oxamic acid**	[c]R132H	<10	K	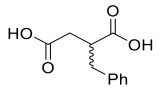 ***DL*-benzylsuccinic acid**	[c]R132H	<10
		[d]R172K	<10			[d]R172K	<10
		[e]R140Q	<10			[e]R140Q	<10
E	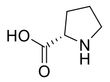 ***L*-proline**	[c]R132H	<10	L	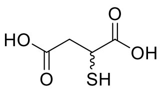 **2-mercaptosuccinic acid**	[c]R132H	<10
		[d]R172K	<10			[d]R172K	<10
		[e]R140Q	<10			[e]R140Q	<10
F	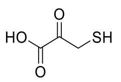 **mercaptopyruvic acid**	[c]R132H	<10	M	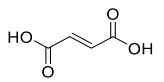 **fumaric acid**	[c]R132H	<10
		[d]R172K	<10			[d]R172K	<10
		[e]R140Q	<10			[e]R140Q	<10
G	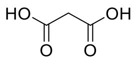 **malonic acid**	[c]R132H	<10	N	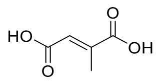 **mesaconic acid**	[c]R132H	<10
		[d]R172K	<10			[d]R172K	<10
		[e]R140Q	<10			[e]R140Q	<10

	[a]2-oxoacids and related small-molecules	IDH1/2 variant	[b]activity [%]		[a]2-oxoacids and related small-molecules	IDH1/2 variant	[b]activity [%]
O	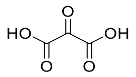 **ketomalonic acid**	[c]R132H	<10	Y	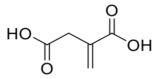 **itaconic acid**	[c]R132H	<10
		[d]R172K	<10			[d]R172K	<10
		[e]R140Q	<10			[e]R140Q	<10
P	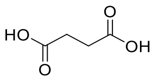 **succinic acid**	[c]R132H	<10	Z	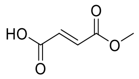 **mono-methyl fumarate**	[c]R132H	<10
		[d]R172K	<10			[d]R172K	<10
		[e]R140Q	<10			[e]R140Q	<10
Q	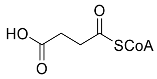 **succinyl CoA**	[c]R132H	<10	AA	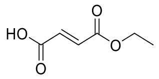 **mono-ethyl fumarate**	[c]R132H	<10
		[d]R172K	<10			[d]R172K	<10
		[e]R140Q	<10			[e]R140Q	<10
R	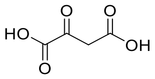 **oxaloacetic acid**	[c]R132H	<10	AB	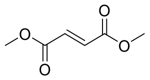 **dimethyl fumarate**	[c]R132H	<10
		[d]R172K	<10			[d]R172K	<10
		[e]R140Q	<10			[e]R140Q	<10
S	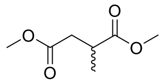 ***DL*-dimethyl 2-methylsuccinate**	[c]R132H	<10	AC	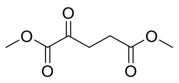 **dimethyl 2-oxoglutaric acid**	[c]R132H	<10
		[d]R172K	<10			[d]R172K	<10
		[e]R140Q	<10			[e]R140Q	<10
T	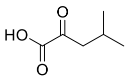 **α-ketoisocaproic acid**	[c]R132H	<10	AD	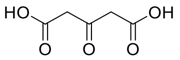 **3-oxoglutaric acid**	[c]R132H	<10
		[d]R172K	<10			[d]R172K	<10
		[e]R140Q	<10			[e]R140Q	<10
U	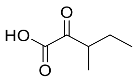 **α-ketoisoleucine**	[c]R132H	<10	AE	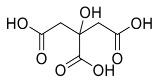 ***DL*-citric acid**	[c]R132H	<10
		[d]R172K	<10			[d]R172K	<10
		[e]R140Q	<10			[e]R140Q	<10
V	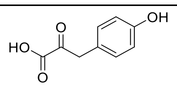 **hydroxyphenylpyruvic acid**	[c]R132H	<10	AF	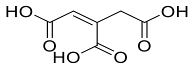 ***cis*-aconitic acid**	[c]R132H	<10
		[d]R172K	<10			[d]R172K	<10
		[e]R140Q	ND			[e]R140Q	<10
W	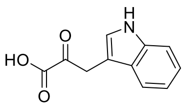 **indole-3-pyruvic acid**	[c]R132H	<10	AG	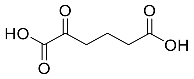 **2-oxoadipic acid**	[c]R132H	<10
		[d]R172K	<10			[d]R172K	<10
		[e]R140Q	ND			[e]R140Q	<10
X	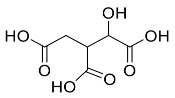 ***DL*-isocitric acid**	[c]R132H	<10	AH	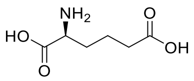 ***L*-2-aminoadipic acid**	[c]R132H	<10
		[d]R172K	<10			[d]R172K	<10
		[e]R140Q	<10			[e]R140Q	<10

	[a]2-oxoacids and related small-molecules	IDH1/2 variant	[b]activity [%]		[a]2-oxoacids and related small-molecules	IDH1/2 variant	[b]activity [%]
AI	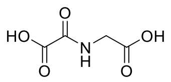 ***N*-oxalylglycine**	[c]R132H	<10	AN	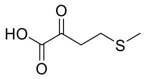 **4-methylthio-2-oxobutyric acid**	[c]R132H	<10
		[d]R172K	<10			[d]R172K	<10
		[e]R140Q	<10			[e]R140Q	<10
AJ	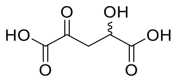 ***DL*-4-hydroxy-2-oxoglutaric acid**	[c]R132H	<10	AO	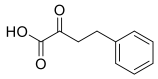 **2-oxo-4-phenylbutanoic acid**	[c]R132H	<10
		[d]R172K	<10			[d]R172K	<10
		[e]R140Q	27 ± 3			[e]R140Q	<10
AK	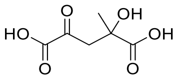 ***DL*-4-hydroxy-4-methyl-2-oxoglutaric acid**	[c]R132H	<10	AP	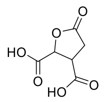 ***DL*-isocitric acid lactone**	[c]R132H	<10
		[d]R172K	<10			[d]R172K	<10
		[e]R140Q	19 ± 2			[e]R140Q	<10
AL	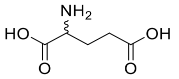 ***DL*-glutamic acid**	[c]R132H	<10	AQ	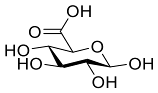 ***D*-glucuronic acid**	[c]R132H	<10
		[d]R172K	<10			[d]R172K	<10
		[e]R140Q	<10			[e]R140Q	<10
AM	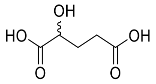 ***DL*-2-hydroglutaric acid**	[c]R132H	<10				
		[d]R172K	<10				
		[e]R140Q	<10				

a) Small-molecules were employed as metal salts in the assay; b) %-substrate conversion after 20 min incubation with IDH1/2 variants; absorbance assays were performed as described in the Experimental Procedures Section; c-e) using 50 µM NADPH and c) 0.03 µM R132H IDH1 and 1.5 mM small-molecule, d) 0.03 µM R172K IDH2 and 1.0 mM small-molecule or e) 0.03 µM R140Q IDH2 and 6.0 mM small-molecule in reaction buffer (100 mM Tris, pH 8.0, 0.005%$_{v/v}$ tween-20, 0.1 mg/mL BSA, 0.2 mM DTT, 10 mM $MgCl_2$,150 mM NaCl); Results are a mean of four technical replicates (n = 4; mean ± SD). ND: not determined due to intrinsic UV absorbance.

Supporting Table S3. 2-Oxoacids and structurally related small-molecules do not inhibit WT IDH1/2 in the presence of equimolar amounts of DL-isocitrate (continues on the following page). The effects of 43 2-oxoacids and structurally related small-molecules, including TCA cycle intermediates, on isolated recombinant human WT IDH1/2 was investigated in the presence of equimolar amounts of DL-isocitrate. No evidence for substantial levels of WT IDH1/2 inhibition (>30%) was accrued.

	[a]2-oxoacids and related small-molecules	WT IDH1/2	[b]activity [%]		[a]2-oxoacids and related small-molecules	WT IDH1/2	[b]activity [%]
A	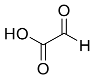 glyoxylic acid	[c]IDH1	104 ± 2	L	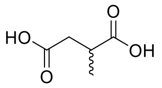 2-methylsuccinic acid	[c]IDH1	103 ± 2
		[d]IDH2	99 ± 2			[d]IDH2	99 ± 3
B	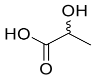 *DL*-lactic acid	[c]IDH1	105 ± 6	M	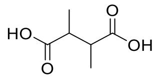 1,2-dimethylsuccinic acid	[c]IDH1	103 ± 1
		[d]IDH2	95 ± 5			[d]IDH2	99 ± 1
C	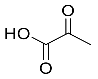 pyruvic acid	[c]IDH1	111 ± 6	N	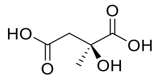 *(R)*-citramalic acid	[c]IDH1	101 ± 1
		[d]IDH2	97 ± 5			[d]IDH2	99 ± 2
D	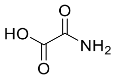 oxamic acid	[c]IDH1	105 ± 2	O	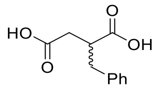 *DL*-benzylsuccinic acid	[c]IDH1	103 ± 4
		[d]IDH2	100 ± 1			[d]IDH2	101 ± 3
E	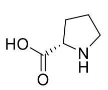 *L*-proline	[c]IDH1	102 ± 1	P	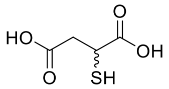 2-mercaptosuccinic acid	[c]IDH1	109 ± 4
		[d]IDH2	98 ± 1			[d]IDH2	100 ± 1
F	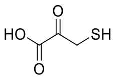 mercaptopyruvic acid	[c]IDH1	88 ± 1	Q	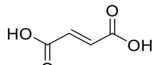 fumaric acid	[c]IDH1	98 ± 1
		[d]IDH2	85 ± 1			[d]IDH2	97 ± 1
G	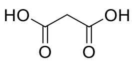 malonic acid	[c]IDH1	100 ± 1	R	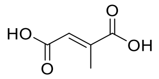 mesaconic acid	[c]IDH1	105 ± 2
		[d]IDH2	100 ± 2			[d]IDH2	100 ± 2
H	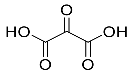 ketomalonic acid	[c]IDH1	102 ± 4	S	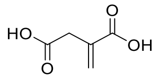 itaconic acid	[c]IDH1	99 ± 1
		[d]IDH2	100 ± 2			[d]IDH2	99 ± 2
I	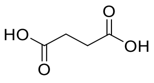 succinic acid	[c]IDH1	97 ± 2	T	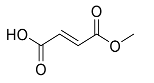 mono-methyl fumarate	[c]IDH1	102 ± 2
		[d]IDH2	97 ± 3			[d]IDH2	98 ± 1
J	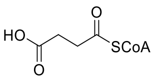 succinyl CoA	[c]IDH1	100 ± 1	U	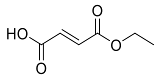 mono-ethyl fumarate	[c]IDH1	106 ± 4
		[d]IDH2	99 ± 1			[d]IDH2	96 ± 5
K	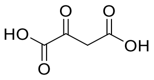 oxaloacetic acid	[c]IDH1	100 ± 1	V	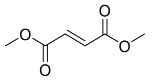 dimethyl fumarate	[c]IDH1	104 ± 4
		[d]IDH2	98 ± 2			[d]IDH2	100 ± 3

	[a]2-oxoacids and related small-molecules	WT IDH1/2	[b]activity [%]		[a]2-oxoacids and related small-molecules	WT IDH1/2	[b]activity [%]
W	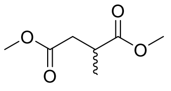 ***DL*-dimethyl 2-methylsuccinate**	[c]IDH1	103 ± 1	AH	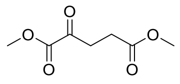 **dimethyl 2-oxoglutaric acid**	[c]IDH1	98 ± 1
		[d]IDH2	100 ± 2			[d]IDH2	97 ± 2
X	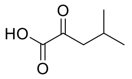 **α-ketoisocaproic acid**	[c]IDH1	102 ± 2	AI	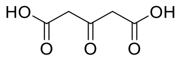 **3-oxoglutaric acid**	[c]IDH1	103 ± 3
		[d]IDH2	102 ± 4			[d]IDH2	99 ± 2
Y	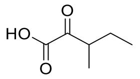 **α-ketoisoleucine**	[c]IDH1	105 ± 4	AJ	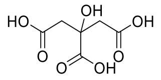 ***DL*-citric acid**	[c]IDH1	103 ± 3
		[d]IDH2	98 ± 3			[d]IDH2	101 ± 3
Z	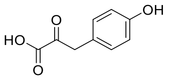 **hydroxyphenyl-pyruvic acid**	[c]IDH1	99 ± 3	AK	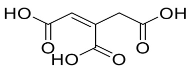 ***cis*-aconitic acid**	[c]IDH1	101 ± 2
		[d]IDH2	96 ± 5			[d]IDH2	99 ± 3
AA	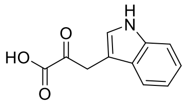 **indole-3-pyruvic acid**	[c]IDH1	88 ± 2	AL	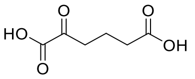 **2-oxoadipic acid**	[c]IDH1	108 ± 8
		[d]IDH2	89 ± 1			[d]IDH2	99 ± 4
AB	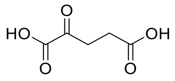 **2-oxoglutaric acid**	[c]IDH1	93 ± 1	AM	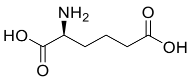 ***L*-2-aminoadipic acid**	[c]IDH1	108 ± 4
		[d]IDH2	91 ± 1			[d]IDH2	101 ± 5
AC	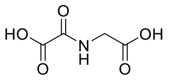 ***N*-oxalylglycine**	[c]IDH1	93 ± 2	AN	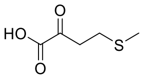 **4-methylthio-2-oxobutyric acid**	[c]IDH1	102 ± 2
		[d]IDH2	93 ± 1			[d]IDH2	98 ± 3
AD	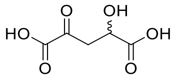 ***DL*-4-hydroxy-2-oxoglutaric acid**	[c]IDH1	89 ± 1	AO	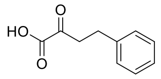 **2-oxo-4-phenylbutanoic acid**	[c]IDH1	94 ± 2
		[d]IDH2	87 ± 1			[d]IDH2	96 ± 6
AE	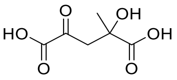 ***DL*-4-hydroxy-4-methyl-2-oxoglutaric acid**	[c]IDH1	101 ± 3	AP	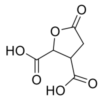 ***DL*-isocitric acid lactone**	[c]IDH1	105 ± 2
		[d]IDH2	99 ± 3			[d]IDH2	106 ± 2
AF	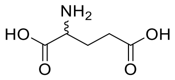 ***DL*-glutamic acid**	[c]IDH1	94 ± 7	AQ	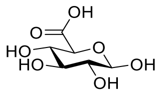 ***D*-glucuronic acid**	[c]IDH1	106 ± 2
		[d]IDH2	99 ± 2			[c]IDH1	99 ± 4
AG	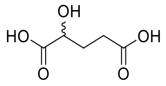 ***DL*-2-hydroglutaric acid**	[c]IDH1	94 ± 2				
		[d]IDH2	95 ± 3				

a) Small-molecules were employed as metal salts in the assay; b) %-substrate conversion after 20 min incubation with WT IDH1/2; absorbance assays were performed as described in the Experimental Procedures Section; c-d) using 75 μM $NADP^+$, 0.15 mM isocitrate, 0.15 mM of the tested small-molecules, and c) 2.0 nM WT IDH1 or d) 2.0 nM WT IDH2, in reaction buffer (100 mM Tris, pH 8.0, $0.005\%_{v/v}$ tween-20, 0.1 mg/mL BSA, 0.2 mM DTT, 10 mM $MgCl_2$, 150 mM NaCl); results are a mean of four technical replicates (n = 4; mean ± SD).

Supporting Table S4. DL-isocitric acid lactone showed evidence for substrate activity with WT IDH1/2 (continues on the following page). The substrate activities of 43 2-oxoacids and structurally related small-molecules, including TCA cycle intermediates, on isolated recombinant human WT IDH1/2 was investigated in the absence of DL-isocitrate. Of the tested 43 small-molecules, only DL-isocitric acid lactone showed evidence for turnover (~10% compared to DL-isocitrate, Entry AP), likely due to hydrolysis.

	[a]2-oxoacids and related small-molecules	WT IDH1/2	[b]activity [%]		[a]2-oxoacids and related small-molecules	WT IDH1/2	[b]activity [%]
A	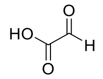 **glyoxylic acid**	[c]IDH1	<10	L	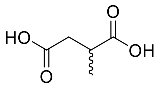 **2-methylsuccinic acid**	[c]IDH1	<10
		[d]IDH2	<10			[d]IDH2	<10
B	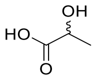 ***DL*-lactic acid**	[c]IDH1	<10	M	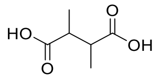 **1,2-dimethylsuccinic acid**	[c]IDH1	<10
		[d]IDH2	<10			[d]IDH2	<10
C	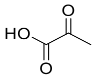 **pyruvic acid**	[c]IDH1	<10	N	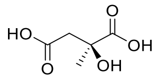 **(*R*)-citramalic acid**	[c]IDH1	<10
		[d]IDH2	<10			[d]IDH2	<10
D	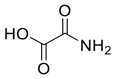 **oxamic acid**	[c]IDH1	<10	O	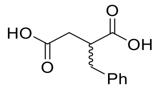 ***DL*-benzylsuccinic acid**	[c]IDH1	<10
		[d]IDH2	<10			[d]IDH2	<10
E	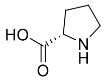 ***L*-proline**	[c]IDH1	<10	P	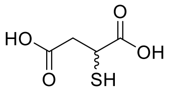 **2-mercaptosuccinic acid**	[c]IDH1	<10
		[d]IDH2	<10			[d]IDH2	<10
F	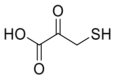 **mercaptopyruvic acid**	[c]IDH1	<10	Q	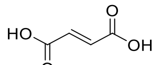 **fumaric acid**	[c]IDH1	<10
		[d]IDH2	<10			[d]IDH2	<10
G	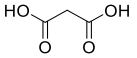 **malonic acid**	[c]IDH1	<10	R	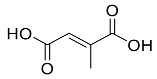 **mesaconic acid**	[c]IDH1	<10
		[d]IDH2	<10			[d]IDH2	<10
H	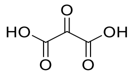 **ketomalonic acid**	[c]IDH1	<10	S	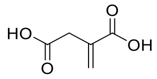 **itaconic acid**	[c]IDH1	<10
		[d]IDH2	<10			[d]IDH2	<10
I	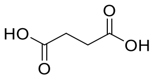 **succinic acid**	[c]IDH1	<10	T	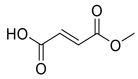 **mono-methyl fumarate**	[c]IDH1	<10
		[d]IDH2	<10			[d]IDH2	<10
J	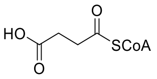 **succinyl CoA**	[c]IDH1	<10	U	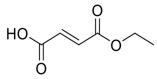 **mono-ethyl fumarate**	[c]IDH1	<10
		[d]IDH2	<10			[d]IDH2	<10
K	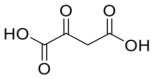 **oxaloacetic acid**	[c]IDH1	<10	V	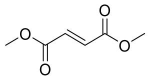 **dimethyl fumarate**	[c]IDH1	<10
		[d]IDH2	<10			[d]IDH2	<10

	[a]2-oxoacids and related small-molecules	WT IDH1/2	[b]activity [%]		[a]2-oxoacids and related small-molecules	WT IDH1/2	[b]activity [%]
W	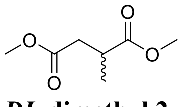 ***DL*-dimethyl 2-methylsuccinate**	[c]IDH1	<10	AH	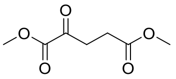 **dimethyl 2-oxoglutaric acid**	[c]IDH1	<10
		[d]IDH2	<10			[d]IDH2	<10
X	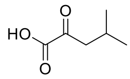 **α-ketoisocaproic acid**	[c]IDH1	<10	AI	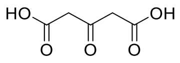 **3-oxoglutaric acid**	[c]IDH1	<10
		[d]IDH2	<10			[d]IDH2	<10
Y	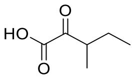 **α-ketoisoleucine**	[c]IDH1	<10	AJ	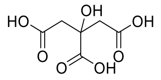 ***DL*-citric acid**	[c]IDH1	<10
		[d]IDH2	<10			[d]IDH2	<10
Z	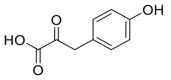 **hydroxyphenyl-pyruvic acid**	[c]IDH1	<10	AK	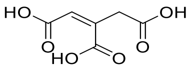 ***cis*-aconitic acid**	[c]IDH1	<10
		[d]IDH2	<10			[d]IDH2	<10
AA	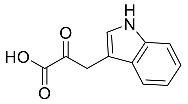 **indole-3-pyruvic acid**	[c]IDH1	<10	AL	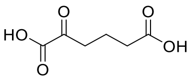 **2-oxoadipic acid**	[c]IDH1	<10
		[d]IDH2	<10			[d]IDH2	<10
AB	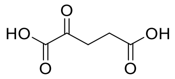 **2-oxoglutaric acid**	[c]IDH1	<10	AM	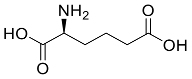 ***L*-2-aminoadipic acid**	[c]IDH1	<10
		[d]IDH2	<10			[d]IDH2	<10
AC	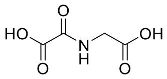 ***N*-oxalylglycine**	[c]IDH1	<10	AN	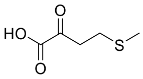 **4-methylthio-2-oxobutyric acid**	[c]IDH1	<10
		[d]IDH2	<10			[d]IDH2	<10
AD	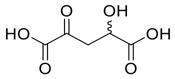 ***DL*-4-hydroxy-2-oxoglutaric acid**	[c]IDH1	<10	AO	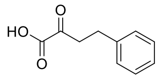 **2-oxo-4-phenylbutanoic acid**	[c]IDH1	<10
		[d]IDH2	<10			[d]IDH2	<10
AE	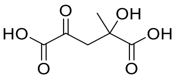 ***DL*-4-hydroxy-4-methyl-2-oxoglutaric acid**	[c]IDH1	<10	AP	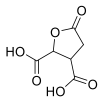 ***DL*-isocitric acid lactone**	[c]IDH1	12 ± 4
		[d]IDH2	<10			[d]IDH2	11 ± 1
AF	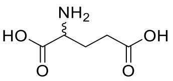 ***DL*-glutamic acid**	[c]IDH1	<10	AQ	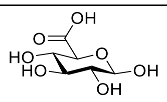 ***D*-glucuronic acid**	[c]IDH1	<10
		[d]IDH2	<10			[c]IDH1	<10
AG	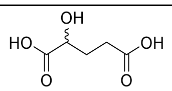 ***DL*-2-hydroglutaric acid**	[c]IDH1	<10				
		[d]IDH2	<10				

a) Small-molecules were employed as metal salts in the assay; b) %-substrate conversion after 20 min incubation with WT IDH1/2; absorbance assays were performed as described in the Experimental Procedures Section; c-d) using 75 µM $NADP^+$, 0.15 mM of the tested small-molecules, and c) 2.0 nM WT IDH1 or d) 2.0 nM WT IDH2, in reaction buffer (100 mM Tris, pH 8.0, $0.005\%_{v/v}$ tween-20, 0.1 mg/mL BSA, 0.2 mM DTT, 10 mM $MgCl_2$, 150 mM NaCl); results are a mean of four technical replicates (n = 4; mean ± SD).

Supporting Table S5. WT IDH1 and WT IDH2 do not accept the tested synthetic 2OG derivatives as substrates (continues on the following page). The substrate activities of 32 synthetic 2OG derivatives (8) with isolated recombinant human WT IDH1 and WT IDH2 was investigated in the absence of DL-isocitrate. None of the tested 2OG derivatives showed substantial levels of substrate activity with WT IDH1 and WT IDH2.

	[a]2OG derivative	WT IDH1/2	[b]activity [%]		[a]2OG derivative	WT IDH1/2	[b]activity [%]
A	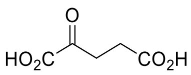 **2OG**	[c]IDH1	<10	L	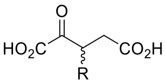 **11**: R = CH_2(9,9-dimethyl-9*H*-fluoren-2-yl)	[c]IDH1	<10
		[d]IDH2	<10			[d]IDH2	<10
B	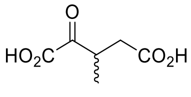 **1**	[c]IDH1	<10	M	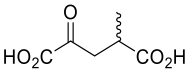 **12**	[c]IDH1	<10
		[d]IDH2	<10			[d]IDH2	<10
C	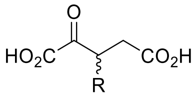 **2**: R = CH_2CH_3	[c]IDH1	<10	N	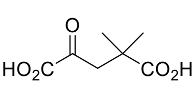 **13**	[c]IDH1	<10
		[d]IDH2	<10			[d]IDH2	<10
D	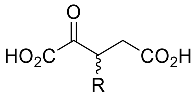 **3**: R = $CH_2CH_2CH_3$	[c]IDH1	<10	O	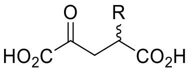 **14**: R = CH_2CH_3	[c]IDH1	<10
		[d]IDH2	<10			[d]IDH2	<10
E	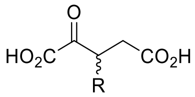 **4**: R = $CH_2CH_2CH_2CH_3$	[c]IDH1	<10	P	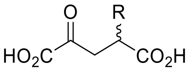 **15**: R = $CH_2CH_2CH_3$	[c]IDH1	<10
		[d]IDH2	<10			[d]IDH2	<10
F	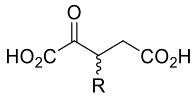 **5**: R = $CH_2CH_2C(CH_3)_3$	[c]IDH1	<10	Q	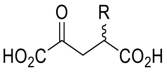 **16**: R = $CH_2CH_2CH_2CH_3$	[c]IDH1	<10
		[d]IDH2	<10			[d]IDH2	<10
G	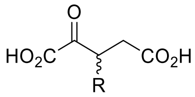 **6**: R = $CH_2CH_2CH_2Ph$	[c]IDH1	<10	R	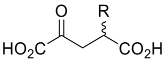 **17**: R = $CH_2CH_2C(CH_3)_3$	[c]IDH1	<10
		[d]IDH2	<10			[d]IDH2	<10
H	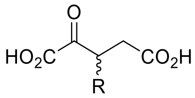 **7**: R = CH_2Ph	[c]IDH1	<10	S	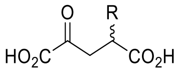 **18**: R = $CH_2CH_2CH_2Ph$	[c]IDH1	<10
		[d]IDH2	<10			[d]IDH2	<10
I	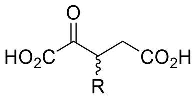 **8**: R = $CH_2(4\text{-}FC_6H_4)$	[c]IDH1	<10	T	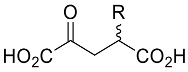 **19**: R = CH_2Ph	[c]IDH1	<10
		[d]IDH2	<10			[d]IDH2	<10
J	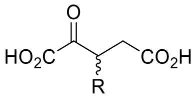 **9**: R = $CH_2(4\text{-}MeOC_6H_4)$	[c]IDH1	<10	U	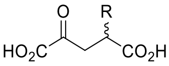 **20**: R = CH_2(2-naphthyl)	[c]IDH1	<10
		[d]IDH2	<10			[d]IDH2	<10
K	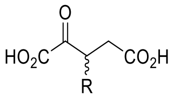 **10**: R = $CH_2(4\text{-}F_3COC_6H_4)$	[c]IDH1	<10	[e]V	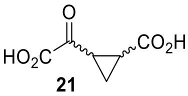 **21**	[c]IDH1	<10
		[d]IDH2	<10			[d]IDH2	<10

	[a]2OG derivative	WT IDH1/2	[b]activity [%]		[a]2OG derivative	WT IDH1/2	[b]activity [%]
[f]W	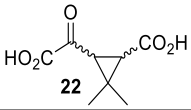 22	[c]IDH1	<10	[j]AC	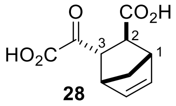 28	[c]IDH1	<10
		[d]IDH2	<10			[d]IDH2	<10
X	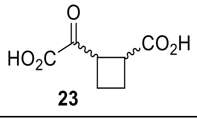 23	[c]IDH1	<10	AD	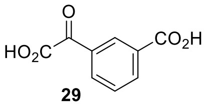 29	[c]IDH1	<10
		[d]IDH2	<10			[d]IDH2	<10
[g]Y	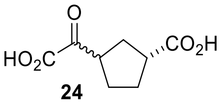 24	[c]IDH1	<10	AE	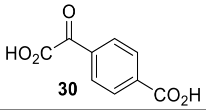 30	[c]IDH1	<10
		[d]IDH2	<10			[d]IDH2	<10
[h]Z	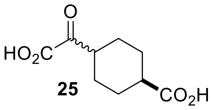 25	[c]IDH1	<10	AF	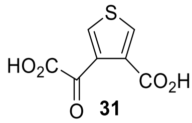 31	[c]IDH1	<10
		[d]IDH2	<10			[d]IDH2	<10
AA	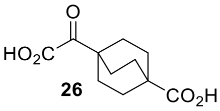 26	[c]IDH1	<10	AG	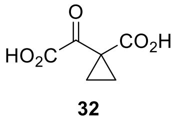 32	[c]IDH1	<10
		[d]IDH2	<10			[c]IDH1	<10
[i]AB	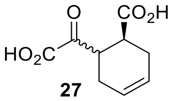 27	[c]IDH1	<10				
		[d]IDH2	<10				

a) 2OG derivatives were prepared from cyanosulfur ylids as racemic mixtures as reported (8); b) %-substrate conversion after 20 min incubation; absorbance assays were performed as described in the Experimental Procedures Section; c-d) using 75 µM $NADP^+$, 0.15 mM of the tested 2OG derivative, and c) 2.0 nM WT IDH1 or d) 2.0 nM WT IDH2, in reaction buffer (100 mM Tris, pH 8.0, $0.005\%_{v/v}$ tween-20, 0.1 mg/mL BSA, 0.2 mM DTT, 10 mM $MgCl_2$, 150 mM NaCl); e) mixture of racemic diastereomers, dr (*cis*:*trans*) = 2.5:1; f) mixture of racemic diastereomers, dr (*cis*:*trans*) = 1:1; g) mixture of racemic diastereomers, dr (*cis*:*trans*) = 2.5:1; h) mixture of diastereomers, dr (*trans*:*cis*) = 5:1; i) mixture of diastereomers, dr (*cis*:*trans*) = 10:1; j) (±)-(*2-exo,3-endo*)-diastereomer. Results are a mean of four technical replicates (n = 4; mean ± SD).

Supporting Table S6. Data collection and refinement statistics for the R132C/S280F IDH1:Ca:NADPH:3-butyl-2OG (4) complex.

	R132C/S280F IDH1:Ca:NADPH:**4** complex
**PDB ID**	8BAY
**Data collection**	
T (in K)	MX (100)
Beamline (Wavelength, Å)	DLS I03 (0.97628)
Detector	Eiger2 XE 16M
Data Processing	Xia2 (9)
Space group	*C* 2 2 2_1
Cell dimensions	
a, *b*, *c* (Å)	99.5, 275.8, 116.6
α, β, γ (°)	90.0, 90.0, 90.0
No. of molecules/ASU	3
No. reflections	67110 (4896)*
Resolution (Å)	58.44-2.35 (2.41-2.35)*
R_{meas} (I)	0.109 (1.890)*
I / σI	14.0 (1.20)*
CC (1/2)	0.999 (0.603)*
Completeness (%)	100 (99.5)*
Multiplicity	11.70 (11.50)*
Wilson B value ($Å^2$)	57.04
**Refinement**	
R_{work} / R_{free}	0.2008/0.2231
No. atoms	10160
Enzyme	9818
Ligand/ion	117
Water	225
Average *B*-factors	67.40
Enzyme ($Å^2$)	67.49
Ligand/ion ($Å^2$)	71.26
Water ($Å^2$)	61.61
R.m.s. deviations	
Bond lengths (Å)	0.099
Bond angles (°)	1.50
Number of TLS groups	10

*Values in parentheses are for highest-resolution shell. DLS = Diamond Light Source.

Supporting Table S7. Steady-state kinetic parameters of the cancer-associated R132C/S280F IDH1 variant for 2OG, 3-methyl-2OG (1), and 3-butyl-2OG (4). The kinetic parameters of R132C/S280F IDH1 for 2OG differ to those obtained for the other tested cancer-associated IDH1/2 variants. The R132C/S280F IDH1 k_{cat}^{app}-value for 2OG is ~3- to ~4-fold higher than that of R132H IDH1, R172K IDH2, and R140Q IDH2 (Entry A). Remarkably, the R132C/S280F IDH1 K_{m}^{app}-value for 2OG is ~10- to ~125-fold lower than that of the other tested IDH1/2 variants, indicating a much higher affinity of R132C/S280F IDH1 for 2OG. Consequently, the R132C/S280F IDH1 k_{cat}/K_{m}-value for 2OG is ~25- to ~580-fold higher than for the other IDH1/2 variants, suggesting it is a better substrate for R132C/S280F IDH1.

The kinetic parameters of R132C/S280F IDH1 for 3-methyl-2OG (**1**) and 3-butyl-2OG (**4**) are similar to those obtained for 2OG; however, both their k_{cat}^{app}-values and K_{m}^{app}-values appeared to be larger, in accord with a similar trend observed for R132H IDH1, R172K IDH2, and R140Q IDH2 (Entries B and C).

	[a]2OG derivative	parameter	R132H IDH1	[b,c]R132C/S280F IDH1	R172K IDH2	R140Q IDH2
A	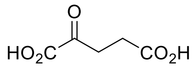 **2OG**	k_{cat}^{app} [s^{-1}]	0.72 ± 0.04	2.3 ± 0.1	0.72 ± 0.04	0.49 ± 0.04
		K_{m}^{app} [mM]	0.85 ± 0.19	0.04 ± 0.01	0.35 ± 0.04	5.1 ± 1.2
		k_{cat}/K_{m} [mM^{-1}·s^{-1}]	0.85 ± 0.22	58 ± 9	2.1 ± 0.3	0.10 ± 0.03
B	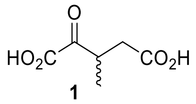 **1**	k_{cat}^{app} [s^{-1}]	6.9 ± 0.4	20.2 ± 0.5	4.8 ± 0.2	5.4 ± 0.3
		K_{m}^{app} [mM]	3.7 ± 0.1	0.31 ± 0.01	4.2 ± 0.4	2.0 ± 0.8
		k_{cat}/K_{m} [mM^{-1}·s^{-1}]	1.9 ± 0.1	65 ± 5	1.2 ± 0.1	2.8 ± 1.1
C	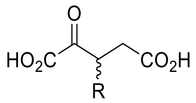 **4**: R = $CH_2CH_2CH_2CH_3$	k_{cat}^{app} [s^{-1}]	9.2 ± 0.4	15 ± 3	7.8 ± 1.8	9.7 ± 0.4
		K_{m}^{app} [mM]	5.7 ± 3.3	0.24 ± 0.01	16 ± 5	3.8 ± 0.8
		k_{cat}/K_{m} [mM^{-1}·s^{-1}]	1.6 ± 1.0	65 ± 15	0.50 ± 0.19	2.6 ± 0.6

a) 2OG derivatives were prepared from cyanosulfur ylids as racemic mixtures as reported (8); b) Mean of two independent runs, each composed of technical duplicates (n = 2; mean ± SD); c) R132C/S280F IDH1 assays monitoring NADPH turnover were performed as reported (3).

Supporting Table S8. C3-Substituted 2OG derivative 11 inhibits oncogenic IDH1/2 variants (continues on the following pages). The inhibitory effects of 32 synthetic 2OG derivatives (8) on isolated recombinant human R132H IDH1, R172K IDH2, and R140Q IDH2 was investigated in the presence of equimolar amounts of 2OG as a substrate. The results reveal that of the tested 2OG derivatives, only **11**, which contains a bulky fluorene-derived substituent at its C3 position, manifests notable inhibition of all three tested IDH1/2 variants (Entry L).

	[a]2OG derivative	IDH1/2 variant	[b]activity [%]		[a]2OG derivative	IDH1/2 variant	[b]activity [%]
A	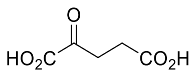 **2OG**	[c]R132H	100	J	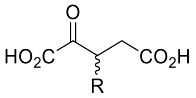 **9**: R = $CH_2(4\text{-}MeOC_6H_4)$	[c]R132H	200 ± 15
		[d]R172K	100			[d]R172K	111 ± 3
		[e]R140Q	100			[e]R140Q	125 ± 3
B	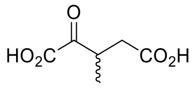 **1**	[c]R132H	391 ± 20	K	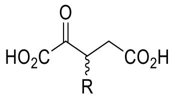 **10**: R = $CH_2(4\text{-}F_3COC_6H_4)$	[c]R132H	108 ± 4
		[d]R172K	151 ± 5			[d]R172K	60 ± 6
		[e]R140Q	107 ± 4			[e]R140Q	159 ± 4
C	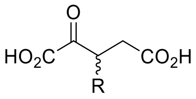 **2**: R = CH_2CH_3	[c]R132H	317 ± 12	L	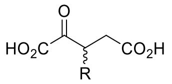 **11**: R = CH_2(9,9-dimethyl-9*H*-fluoren-2-yl)	[c]R132H	<10
		[d]R172K	131 ± 16			[d]R172K	<10
		[e]R140Q	865 ± 78			[e]R140Q	<10
D	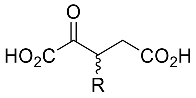 **3**: R = $CH_2CH_2CH_3$	[c]R132H	294 ± 31	M	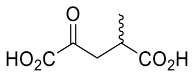 **12**	[c]R132H	161 ± 26
		[d]R172K	105 ± 6			[d]R172K	112 ± 13
		[e]R140Q	632 ± 68			[e]R140Q	281 ± 4
E	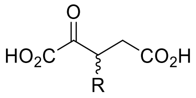 **4**: R = $CH_2CH_2CH_2CH_3$	[c]R132H	376 ± 21	N	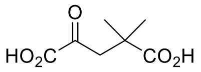 **13**	[c]R132H	118 ± 20
		[d]R172K	126 ± 3			[d]R172K	106 ± 6
		[e]R140Q	753 ± 72			[e]R140Q	117 ± 6
F	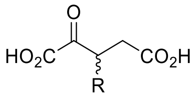 **5**: R = $CH_2CH_2C(CH_3)_3$	[c]R132H	142 ± 14	O	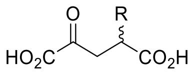 **14**: R = CH_2CH_3	[c]R132H	103 ± 2
		[d]R172K	98 ± 3			[d]R172K	112 ± 13
		[e]R140Q	146 ± 4			[e]R140Q	117 ± 9
G	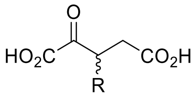 **6**: R = $CH_2CH_2CH_2Ph$	[c]R132H	182 ± 10	P	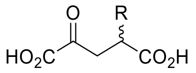 **15**: R = $CH_2CH_2CH_3$	[c]R132H	101 ± 7
		[d]R172K	93 ± 1			[d]R172K	97 ± 5
		[e]R140Q	239 ± 37			[e]R140Q	116 ± 4
H	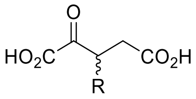 **7**: R = CH_2Ph	[c]R132H	229 ± 21	Q	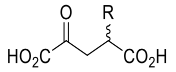 **16**: R = $CH_2CH_2CH_2CH_3$	[c]R132H	113 ± 4
		[d]R172K	114 ± 4			[d]R172K	100 ± 5
		[e]R140Q	91 ± 8			[e]R140Q	131 ± 9
I	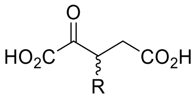 **8**: R = $CH_2(4\text{-}FC_6H_4)$	[c]R132H	186 ± 6	R	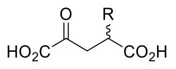 **17**: R = $CH_2CH_2C(CH_3)_3$	[c]R132H	93 ± 3
		[d]R172K	106 ± 5			[d]R172K	93 ± 3
		[e]R140Q	516 ± 40			[e]R140Q	119 ± 5

	[a]2OG derivative	IDH1/2 variant	[b]activity [%]		[a]2OG derivative	IDH1/2 variant	[b]activity [%]
S	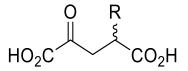 **18**: R = $CH_2CH_2CH_2Ph$	[c]R132H	82 ± 3	AA	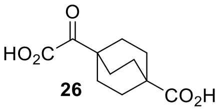 **26**	[c]R132H	92 ± 3
		[d]R172K	74 ± 12			[d]R172K	102 ± 5
		[e]R140Q	101 ± 3			[e]R140Q	99 ± 2
T	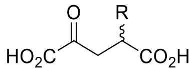 **19**: R = CH_2Ph	[c]R132H	116 ± 17	[j]AB	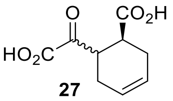 **27**	[c]R132H	94 ± 11
		[d]R172K	102 ± 5			[d]R172K	104 ± 8
		[e]R140Q	106 ± 4			[e]R140Q	97 ± 4
U	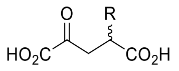 **20**: R = CH_2(2-naphthyl)	[c]R132H	31 ± 2	[k]AC	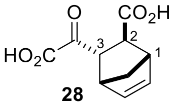 **28**	[c]R132H	116 ± 4
		[d]R172K	51 ± 1			[d]R172K	105 ± 5
		[e]R140Q	73 ± 4			[e]R140Q	114 ± 4
[f]V	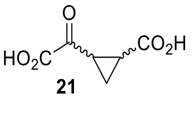 **21**	[c]R132H	132 ± 3	AD	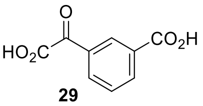 **29**	[c]R132H	88 ± 2
		[d]R172K	110 ± 4			[d]R172K	93 ± 7
		[e]R140Q	394 ± 40			[e]R140Q	95 ± 7
[g]W	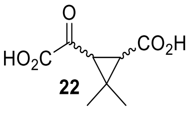 **22**	[c]R132H	92 ± 5	AE	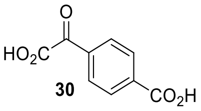 **30**	[c]R132H	103 ± 2
		[d]R172K	110 ± 10			[d]R172K	110 ± 8
		[e]R140Q	112 ± 4			[e]R140Q	94 ± 3
X	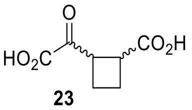 **23**	[c]R132H	193 ± 16	AF	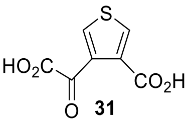 **31**	[c]R132H	102 ± 5
		[d]R172K	122 ± 6			[d]R172K	99 ± 3
		[e]R140Q	260 ± 87			[e]R140Q	113 ± 4
[h]Y	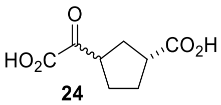 **24**	[c]R132H	99 ± 9	AG	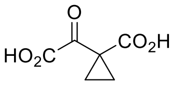 **32**	[c]R132H	88 ± 2
		[d]R172K	110 ± 2			[d]R172K	94 ± 6
		[e]R140Q	113 ± 4			[e]R140Q	105 ± 4
[i]Y	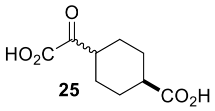 **25**	[c]R132H	105 ± 6				
		[d]R172K	71 ± 13				
		[e]R140Q	ND				

a) 2OG derivatives were prepared from cyanosulfur ylids as racemic mixtures as reported (8); b) %-substrate conversion after 20 min incubation; absorbance assays were performed as described in the Experimental Procedures Section; c-e) using 50 µM NADPH and c) 0.03 µM R132H IDH1, 1.5 mM 2OG, and 1.5 mM 2OG derivative, d) 0.03 µM R172K IDH2, 1.0 mM 2OG, and 1.0 mM 2OG derivative or e) 0.03 µM R140Q IDH2, 6.0 mM 2OG, and 6.0 mM 2OG derivative in reaction buffer (100 mM Tris, pH 8.0, 0.005%$_{v/v}$ tween-20, 0.1 mg/mL BSA, 0.2 mM DTT, 10 mM $MgCl_2$,150 mM NaCl); f) mixture of racemic diastereomers, dr (*cis*:*trans*) = 2.5:1; g) mixture of racemic diastereomers, dr (*cis*:*trans*) = 1:1; h) mixture of racemic diastereomers, dr (*cis*:*trans*) = 2.5:1; i) mixture of diastereomers, dr (*trans*:*cis*) = 5:1; j) mixture of diastereomers, dr (*cis*:*trans*) = 10:1; k) (±)-(*2-exo,3-endo*)-diastereomer. Results are a mean of four technical replicates (n = 4; mean ± SD). ND: not determined due to intrinsic UV absorbance.

Supporting Table S9. The tested synthetic 2OG derivatives do not inhibit WT IDH1/2 (continues on the following page). The inhibitory effects of 32 synthetic 2OG derivatives (8) on isolated recombinant human WT IDH1/2 was investigated using absorbance assays. None of the tested 2OG derivatives showed substantial levels of WT IDH1/2 inhibition.

	[a]2OG derivative	WT IDH1/2	[b]activity [%]		[a]2OG derivative	WT IDH1/2	[b]activity [%]
A	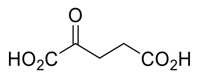 **2OG**	[c]IDH1	93 ± 1	L	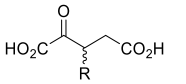 **11**: R = CH_2(9,9-dimethyl-9*H*-fluoren-2-yl)	[c]IDH1	98±2
		[d]IDH2	91 ± 1			[d]IDH2	96±2
B	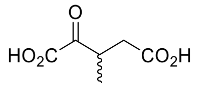 **1**	[c]IDH1	94±1	M	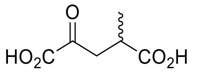 **12**	[c]IDH1	107±4
		[d]IDH2	97±3			[d]IDH2	100±4
C	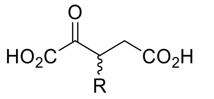 **2**: R = CH_2CH_3	[c]IDH1	98±10	N	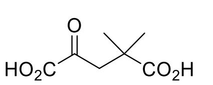 **13**	[c]IDH1	106±4
		[d]IDH2	96±3			[d]IDH2	101±7
D	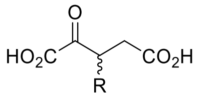 **3**: R = $CH_2CH_2CH_3$	[c]IDH1	105±5	O	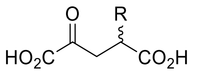 **14**: R = CH_2CH_3	[c]IDH1	94±1
		[d]IDH2	96±5			[d]IDH2	98±3
E	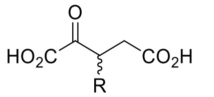 **4**: R = $CH_2CH_2CH_2CH_3$	[c]IDH1	96±1	P	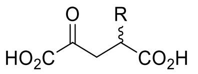 **15**: R = $CH_2CH_2CH_3$	[c]IDH1	95±3
		[d]IDH2	98±2			[d]IDH2	98±3
F	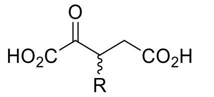 **5**: R = $CH_2CH_2C(CH_3)_3$	[c]IDH1	95±6	Q	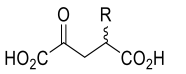 **16**: R = $CH_2CH_2CH_2CH_3$	[c]IDH1	102±3
		[d]IDH2	98±1			[d]IDH2	100±2
G	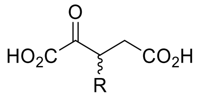 **6**: R = $CH_2CH_2CH_2Ph$	[c]IDH1	96±1	R	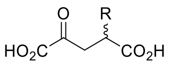 **17**: R = $CH_2CH_2C(CH_3)_3$	[c]IDH1	95±7
		[d]IDH2	99±1			[d]IDH2	100±2
H	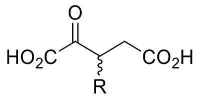 **7**: R = CH_2Ph	[c]IDH1	94±1	S	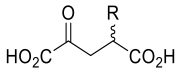 **18**: R = $CH_2CH_2CH_2Ph$	[c]IDH1	101±3
		[d]IDH2	99±1			[d]IDH2	100±1
I	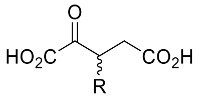 **8**: R = $CH_2(4\text{-}FC_6H_4)$	[c]IDH1	96±3	T	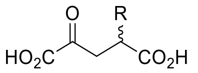 **19**: R = CH_2Ph	[c]IDH1	98±2
		[d]IDH2	100±1			[d]IDH2	97±3
J	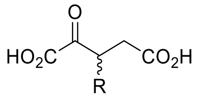 **9**: R = $CH_2(4\text{-}MeOC_6H_4)$	[c]IDH1	93±2	U	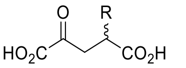 **20**: R = CH_2(2-naphthyl)	[c]IDH1	97±3
		[d]IDH2	98±3			[d]IDH2	97±4
K	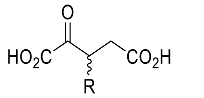 **10**: R = $CH_2(4\text{-}F_3COC_6H_4)$	[c]IDH1	100±2	[e]V	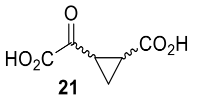 **21**	[c]IDH1	96±1
		[d]IDH2	100±1			[d]IDH2	95±2

	[a]2-oxoacids and related small-molecules	WT IDH1/2	[b]activity [%]		[a]2-oxoacids and related small-molecules	WT IDH1/2	[b]activity [%]
[f]W	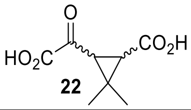 22	[c]IDH1	96±1	[j]AC	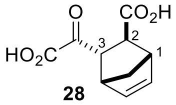 28	[c]IDH1	97±1
		[d]IDH2	96±1			[d]IDH2	99±1
X	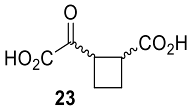 23	[c]IDH1	100±3	AD	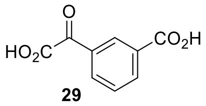 29	[c]IDH1	94±2
		[d]IDH2	99±4			[d]IDH2	99±1
[g]Y	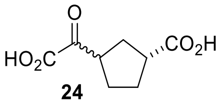 24	[c]IDH1	104±7	AE	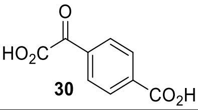 30	[c]IDH1	101±2
		[d]IDH2	100±2			[d]IDH2	100±4
[h]Z	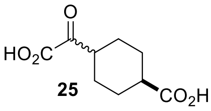 25	[c]IDH1	104±6	AF	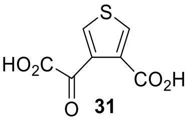 31	[c]IDH1	103±1
		[d]IDH2	92±9			[d]IDH2	101±2
AA	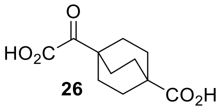 26	[c]IDH1	99±3	AG	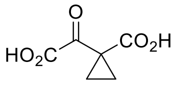 32	[c]IDH1	95±3
		[d]IDH2	100±2			[c]IDH1	97±3
[i]AB	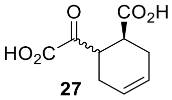 27	[c]IDH1	93±1				
		[d]IDH2	92±4				

a) 2OG derivatives were prepared from cyanosulfur ylids as racemic mixtures as reported (8); b) %-substrate conversion after 20 min incubation; absorbance assays were performed as described in the Experimental Procedures Section; c-d) using 75 µM $NADP^+$, 0.15 mM of DL-isocitrate, 0.15 mM of the tested 2OG derivative, and c) 2.0 nM WT IDH1 or d) 2.0 nM WT IDH2, in reaction buffer (100 mM Tris, pH 8.0, 0.005%$_{v/v}$ tween-20, 0.1 mg/mL BSA, 0.2 mM DTT, 10 mM $MgCl_2$, 150 mM NaCl); f) mixture of racemic diastereomers, dr (*cis*:*trans*) = 2.5:1; f) mixture of racemic diastereomers, dr (*cis*:*trans*) = 1:1; g) mixture of racemic diastereomers, dr (*cis*:*trans*) = 2.5:1; h) mixture of diastereomers, dr (*trans*:*cis*) = 5:1; i) mixture of diastereomers, dr (*cis*:*trans*) = 10:1; j) (±)-(*2-exo,3-endo*)-diastereomer. Results are a mean of four technical replicates (n = 4; mean ± SD).

6. References

1. Herold, R. A., Reinbold, R., Megarity, C. F., Abboud, M. I., Schofield, C. J., and Armstrong, F. A. (2021) Exploiting electrode nanoconfinement to investigate the catalytic properties of isocitrate dehydrogenase (IDH1) and a cancer-associated variant. *J. Phys. Chem. Lett.* **12**, 6095-6101
2. Aguilar, J. A., Nilsson, M., Bodenhausen, G., and Morris, G. A. (2012) Spin echo NMR spectra without J modulation. *Chem. Commun.* **48**, 811-813
3. Reinbold, R., Hvinden, I. C., Rabe, P., Herold, R. A., Finch, A., Wood, J., Morgan, M., Staudt, M., Clifton, I. J., Armstrong, F. A., McCullagh, J. S. O., Redmond, J., Bardella, C., Abboud, M. I., and Schofield, C. J. (2022) Resistance to the isocitrate dehydrogenase 1 mutant inhibitor ivosidenib can be overcome by alternative dimer-interface binding inhibitors. *Nat. Commun.* **13**, 4785
4. Adams, E., and Frank, L. (1980) Metabolism of proline and the hydroxyprolines. *Annu. Rev. Biochem.* **49**, 1005-1061
5. Riedel, T. J., Knight, J., Murray, M. S., Milliner, D. S., Holmes, R. P., and Lowther, W. T. (2012) 4-Hydroxy-2-oxoglutarate aldolase inactivity in primary hyperoxaluria type 3 and glyoxylate reductase inhibition. *Biochim. Biophys. Acta* **1822**, 1544-1552
6. Grobbelaar, N., Pollard, J. K., and Steward, F. C. (1955) New soluble nitrogen compounds (amino- and imino-acids and amides) in plants. *Nature* **175**, 703-708
7. Mazurkewich, S., Brott, A. S., Kimber, M. S., and Seah, S. Y. K. (2016) Structural and kinetic characterization of the 4-carboxy-2-hydroxymuconate hydratase from the gallate and protocatechuate 4,5-cleavage pathways of Pseudomonas putida KT2440. *J. Biol. Chem.* **291**, 7669-7686
8. Brewitz, L., Nakashima, Y., and Schofield, C. J. (2021) Synthesis of 2-oxoglutarate derivatives and their evaluation as cosubstrates and inhibitors of human aspartate/asparagine-β-hydroxylase. *Chem. Sci.* **12**, 1327-1342
9. Winter, G. (2010) xia2: an expert system for macromolecular crystallography data reduction. *J. Appl. Cryst.* **43**, 186-190
